# Association Study of the Caspase Gene Family and Psoriasis Vulgaris Susceptibility in Northeastern China

**DOI:** 10.1155/2019/2417612

**Published:** 2019-02-17

**Authors:** Xinyu Yao, Siyu Hao, Pei Yu

**Affiliations:** ^1^Department of Dermatology, Peking University First Hospital, Beijing, 100034, China; ^2^Department of Dermatology, The Second Affiliated Hospital of Harbin Medical University, Harbin, 150081, China

## Abstract

**Background:**

Abnormal apoptosis of keratinocytes is one of the pathological changes of psoriasis. Caspases (CASPs) are the central engines of apoptosis. Studies to date have shown that some SNPs alter the expression of related genes and lead to changes in disease risk. However, no studies have investigated the associations between gene polymorphisms and the risk of psoriasis in Han population in northeast China. Therefore, we conducted a case-control study to explore this question in Han population of northeastern China.

**Methods:**

540 patients with PsV and 612 healthy age- and sex-matched controls were enrolled in this study. We determined the genotypes of 17 single nucleotide polymorphisms (SNPs) from 11 genes of caspase family by the improved multiplex ligation detection reaction (iMLDR) method. A model-based single SNP frequentist test and haplotype association studies were performed to evaluate the association between SNPs and PsV.

**Results:**

In the single SNP tests, rs6704688 in* CASP8* was significantly associated with psoriasis vulgaris (PsV) in Han population of northeastern China (P = 0.0169, P' = 0.0179 under the additive model;* P* = 0.0126,* P'* = 0.0149 under the heterozygous model). In haplotype analyses, the* CASP7* haplotype GC was found to be associated with PsV risk (case group versus control group, 47.2% versus 54.4%, respectively,* p* = 0.0149).

**Conclusions:**

Our study presented that the gene polymorphisms of* CASP7* and* CASP8* were significantly associated with PsV in Han population of northeastern China, which implied the functional relationship between PsV and caspase genes.* CASP8* and* CASP7* SNPs could be new potential biomarkers for risk stratification and prevention of PsV.

## 1. Introduction

Psoriasis is a common chronic relapsing inflammatory skin disorder that is induced and sustained by lymphocytes infiltrating the skin, affecting about 2-3% of the population [1, 2]. Although its etiology is not clear, we know that it is not only a skin-restricted disease but also a systemic inflammatory immune disorder which is influenced by various genetic and environmental factors [3]. As with many autoimmune diseases, abnormal apoptosis has important implications in psoriasis biology [4, 5]. The data show that, in addition to the hyperproliferation of keratinocytes, deceleration in keratinocyte apoptosis is also a significant pathological change observed in psoriasis [6–8]. Keratinocytes from the lesion of psoriasis are resistant to induction of apoptosis compared to keratinocytes from normal skin [9]. However, the current data on the apoptosis of psoriatic keratinocytes is limited. Therefore, there is a need to better understand the mechanisms of apoptosis in the progression of psoriasis and to identify potential biomarkers for prognostic prediction.

Apoptosis, a unique physiological process genetically controlling programmed cell death, is essential for maintaining normal tissue homeostasis, cell differentiation, and development [4]. In the skin, the death of apoptotic cells regulates keratinocyte proliferation and the formation of the stratum corneum [10]. Caspases (CASPs) are cysteine-dependent aspartate-specific proteases that play a central role in the induction, transduction, and amplification of apoptotic signals in cells and thus determination of cell fate [11, 12]. CASPs can be generally classified into three groups based on their biological functions: inflammatory CASPs (CASP1, CASP4, CASP15, CASP11, and CASP12, apoptosis initiator (CASP2, CASP8, CASP9, and CASP10), and apoptosis executioner (CASP3, CASP6, and CASP7). CASP14 plays an important role in the terminal differentiation of epidermal keratinocytes [13]. The apoptotic pathways involved in keratinocyte apoptosis include several mechanisms. But all have to go through the caspase family. CASPs are sequentially activated in apoptosis, and any CASP may lead to apoptosis aberration. The increase of epidermal thickness in psoriasis may be related to the abnormality of the apoptotic pathway [2]. Some of them have even been reported to have abnormal expression in the lesions of psoriasis [1, 14, 15].

Psoriasis is a widespread autoimmune disease and systemic inflammatory disorder with which many of comorbidities (e.g., diabetes, cardiovascular diseases, Crohn's disease, lymphoma, and cancer) are associated. Patients with psoriasis tend to have an increased risk of cancer [16]. Single nucleotide polymorphisms (SNPs) influence the expression or the activities of caspase enzymes. Several* CASP* genes polymorphisms have been found to be associated with kinds of cancers [17–21] and autoimmune inflammatory diseases, such as rheumatoid arthritis [5], coronary artery disease [22], Kawasaki disease [23], acute pancreatitis [24], and ischemic stroke [25], but the relationship with psoriasis has not been studied. And gene expression profiles showed significant differences in the expression of CASP7 and CASP8 between psoriatic lesions and psoriatic nonlesions. With this in mind, we designed a case-control trial to explore the relationship between* CASP* genes and psoriasis vulgaris (PsV) using polymorphism association studies in order to elucidate the pathogenic mechanisms of PsV.

## 2. Materials and Methods

### 2.1. Study Population

The present case-control study included 540 patients with PsV randomly enrolled from the Second Affiliated Hospital of Harbin Medical University from January 2013 to April 2017 and 612 healthy controls randomly recruited from the same hospital during routine health examinations performed during the same period. All samples we collected were between the ages of 5 and 85. The patients with PsV were diagnosed by at least two clinical dermatologists in the Department of Dermatology. All subjects were of Han Chinese descent in northeastern China; all patients underwent a standardized clinical evaluation and the healthy controls were without any known history of PsV (including three levels of relatives).

The basic information of all subjects including sex, age, and demographic characteristics (in the case group), including age of onset, severity grade, and family history (including three levels of relatives), was obtained from self-reported questionnaires and medical records. Subjects with systemic, infectious, autoimmune, atopic, or malignant disease were excluded.

All of the participants provided written informed consent before participating in the study. All the procedures followed were in accordance with the ethical standards of the 2nd Affiliated Hospital of Harbin Medical University on human experimentation. The Ethics Committees of the 2nd Affiliated Hospital of Harbin Medical University approved this study. All procedures were in accordance with the 1975 Declaration of Helsinki and its later amendments.

### 2.2. DNA Extraction

Genomic DNA was prepared from 2 mL of intravenous whole blood using the QIAamp DNA Blood Mini Kit (Qiagen, Hilden, Germany) according to the manufacturer's instructions. DNA samples were stored in a freezer (Mitsubishi, Japan) at −20°C.

### 2.3. Selection of Polymorphisms

Seventeen polymorphisms in 11* CASP* genes were examined. We did not find any SNPs in* CASP2* with the appropriate allele frequency in Han population of northeastern China.* CASP11*,* CASP13*, and* CASP15 *do not exist in humans. For the 17 common SNPs, 10 polymorphisms (*CASP3* rs2705897 and rs4647610,* CASP5* rs507879,* CASP7* rs2227310,* CASP8* rs6704688 and rs2293554,* CASP9* rs4233532 and rs1052576, and* CASP10* rs12613347 and rs13006529) were selected based on the literature suggesting that these SNPs were associated with the risk of various cancers and autoimmune diseases. For* CASP1*,* CASP4*,* CASP6*,* CASP7*,* CASP12*, and* CASP14*, we utilized the International HapMap Project database (http://hapmap.ncbi.nlm.nih.gov/) and the NCBI dbSNP database (https://www.ncbi.nlm.nih.gov/projects/SNP/) to select potentially functional SNPs. We set the cutoff at a minor allele frequency of more than 0.05 for the Han Chinese in Beijing (HCB) population and linkage disequilibrium (LD) patterns with r^2^ values of more than 0.8. Based on the different distributions of SNPs, seven representative common SNPs (*CASP1* rs2282659,* CASP4* rs547584 and rs672016,* CASP6* rs5030545,* CASP7* rs17090911,* CASP12* rs506601, and* CASP14* rs3181304) were finally selected for genotyping.

### 2.4. Genotyping

Genotyping was performed by the Improved Multiple Ligase Detection Reaction technique developed by Genesky Biotechnologies Inc. (Shanghai, China) [26]. Target DNA sequences were amplified using a multiplex polymerase chain reaction (PCR) method with specific primers and probes. The details are shown in Supplementary Tables S1 and S2.

Data analysis was carried out using GeneMapper Software version 4.1. For quality control, genotyping was carried out without knowledge of group status. We randomly selected about 10% of samples for further verification and obtained a concordance rate of 100%.

### 2.5. Statistics

The demographic characteristics were calculated by SPSS version 19.0 (IBM, Chicago, IL, USA). *χ*^2^ tests and Student's t-tests were used to compare clinical characteristics between the two groups. The Hardy-Weinberg equilibrium (HWE; evaluated at < 0.001) test was determined using *χ*^2^ tests. Associations between candidate SNPs and PsV risk were determined based on the distributions of allele and genotypic frequencies along with the additive, dominant, recessive, and heterozygous models. Multiple logistic regressions analyses were used to analyze the genotype frequency between groups. The gene interaction analysis (gene epistasis analysis) was performed using the Epistasis Module of Plink. Odds ratios and 95% confidence intervals were calculated to determine the relative risk of PsV, and 10,000 permutations were performed for the multiple test correction. The LD and haplotype were computed and constructed from the genotypes of these polymorphic markers by Haploview 4.0, and LD r^2^ values were limited to 0.8. All comparisons were two sided, and results with *P* values of less than 0.05 were considered statistically significant.

## 3. Results

### 3.1. Participants

Basic information and the clinical characteristics of the participants are shown in Table 1. No significant differences were observed in these factors between the two groups.

### 3.2. Single SNP Analysis

The genotype and allele frequencies of the SNPs in the PsV group and control group are shown in Tables 2 and 3. All genotype distributions of cases and controls passed the H-W test. For the* CASP8* rs6704688 polymorphism, the major C allele (risk allele) was present in 76.9% of patients and 75.3% of controls. The additive model (C versus T) distribution and the heterozygous model (TT + CC versus TC) distribution were significantly different between cases and controls (*P* = 0.0169,* P'* = 0.0179;* P* = 0.0126, and* P'* = 0.0149, respectively). No associations between the other 16 SNPs and the risk of PsV were observed in this study. However, there were correlations among* CASP8* rs6704688 in the dominant model (CC versus CT + TT;* P* = 0.0679,* P'* = 0.0974),* CASP9* rs4233532 in the heterozygous model (CC + TT versus CT;* P* = 0.0773,* P'* = 0.0821),* CASP10* rs12613347 in the recessive model (CC + CT versus TT;* P* = 0.8247,* P'* = 0.8681) and heterozygous model (TT + CC versus TC;* P* = 0.8829,* P'* = 0.9316), and* CASP10* rs13006529 in the additive model (T versus A;* P* = 0.0846,* P'* = 0.0806). These results were marginally significant with regard to PsV risk and may become significant if an increased sample size is evaluated.

### 3.3. Interaction Analysis

We performed a gene-gene interaction analysis on all samples. We found that the interaction of* CASP10* -* CASP1, CASP10*-* CASP3, CASP10*-* CASP4, CASP10*-* CASP9, CASP10*-* CASP12, CASP8 - CASP3, CASP8 - CASP4*, and* CASP1 - CASP12* is important for the risk of psoriasis (Table 4). Additional results that were not found to be significant were shown in Supplementary Materials (Table S3). The age group under 40 is a high-risk age group for psoriasis. The analysis of intergene interactions in this age group found that the interaction between 10 pairs of genes was significant for the risk of psoriasis. They are the interaction of* CASP8* -*CASP10*,* CASP3* -* CASP4*,* CASP3 - CASP6, CASP3*-*CASP8*,* CASP1* -* CASP8, CASP1*-* CASP10*,* CASP6* -* CASP7*, and* CASP7*-* CASP9* (Table 5). Additional results that were not found to be significant were shown in Supplementary Materials (Table S4).

### 3.4. Haplotype Analysis

The haplotype blocks were imputed by Haploview for each of the 11 genes and we obtained four haplotypes in* CASP3* and* CASP4* and three haplotypes in* CASP7*,* CASP8*,* CASP9*, and* CASP10*. The results are shown in Table 6. The results showed that, among the 21 haplotypes, the* CASP3* haplotype TT and* CASP4* haplotype TC were the two most common in the case and control groups (TT frequencies of 0.633 and 0.657, respectively, and TC frequencies of 0.619 and 0.643, respectively). The* CASP7* haplotype GC was significantly different in patients with PsV than in controls (47.2% versus 54.4%,* p* = 0.0149). We did not identify the other haplotypes to be significantly associated with PsV (*P* > 0.05).

We also used Haploview to calculate the haplotypes of individual genes on the same chromosome, estimated the haplotype frequencies, and compared the haplotype distribution between the patients and control groups. We found that the haplotype frequency of* CASP3* (rs2705897) on chromosome 4,* CASP7 *(rs17090911) on chromosome 10, CASP10 (rs12613347) on chromosome 2, and* CASP12*(rs506601) on chromosome 11 was significantly different between the patients group and the control group. The results are shown in Table 7. We performed the same analysis for the age group under 40 years old and found that the haplotype frequency of* CASP7* (rs17090911) on chromosome 10,* CASP9 *(rs4233532) on chromosome 1,* CASP10* (rs12613347) on chromosome 2, and* CASP12 *(rs506601) on chromosome 11 was significantly different between the patients group and the control group. The results are shown in Table 8.

## 4. Discussion

Genome-wide association studies (GWASs) have identified numerous single nucleotide polymorphisms (SNPs) associated with psoriasis risk [27, 28]; however, our study was the first to evaluate the association of* CASP* family genes SNPs with risk of PsV in Han population in northeast China. We found that* CASP8* rs6704688 and the* CASP7 *haplotype GC were significantly different between cases and controls. These findings suggested that polymorphisms in* CASP7 *and* CASP8* were associated with PsV risk in Han population of northeastern China and provide new insights into the importance of* CASP7* and* CASP8* in psoriasis susceptibility.

The apoptotic pathways of keratinocytes mainly include two mechanisms, and the process is regulated by a variety of proteins, genes, and internal and external stimuli. The “extrinsic" pathway is triggered by the binding of Fas ligand (FasL) or tumor necrosis factor (TNF) to membrane death receptors and these membrane death receptors recruit adapter molecules resulting in the activation of CASP8 [10]. The activated CASP8 then initiates a downstream apoptotic cascade by cleaving CASP3 and/or CASP7 leading to apoptosis. The “intrinsic” pathway includes mitochondrial release of cytochrome c and, along with the cofactor Apaf-1, the formation of an activated CASP9 apoptosome [10]. CASP3 and/or CASP7 are then activated, ultimately leading to apoptosis. Finally, both pathways end with DNA cleavage, which then forms apoptotic bodies and is phagocytosed by neighboring cells.

Human* CASP7 *is located on chromosome 10q25.3 and contains 13 exons, and human* CASP8* is located on chromosome 2q33.1 and contains 14 exons. GWAS study suggests that there are significant correlations between SNPs in the vicinity of* CASP7* and* CASP8* and psoriasis (the details are shown in Supplementary Tables S5 and S6.)* CASP7* and* CASP8 *encode CASP7 and CASP8 proteins, respectively. SNPs can strongly influence plasma levels and the biological activities of their corresponding proteins [29]. A six-nucleotide insertion/deletion variant rs3834129, which is located in the* CASP8* promoter region, is associated with decreased risk of various cancers including cutaneous melanoma [30–32]. rs6704688 is located in the intron region of* CASP8*. While the function of rs6704688 is not yet known, we know that it is located near rs3834129 and is in high LD with rs3834129. Therefore, we inferred that rs6704688 may not be a true causal variant and that the relationship between rs6704688 and PsV could be attributed to the presence of rs3834129 in the population. Computational molecular and biochemical analyses have confirmed that the rs3834129 DEL allele disrupts the binding site of the transcriptional activator Sp1, thereby reducing the transcription and expression of* CASP8* [31]. Low CASP8 expression or functional aberrations may suppress T-lymphocyte apoptosis [31]. T cells and their cytokines play a central role in the pathogenesis of psoriasis [33]. The pathogenic T cells enter the skin, become activated, and release chemokines and cytokines to attract other immune cells to mediate keratinocyte hyperproliferation and maintain the inflammatory process of psoriasis [33]. Studies have shown the DEL allele and DEL carrier of rs3834129 had negative associations with cancer susceptibility in Asian populations and rs6704688 is associated with reduced cancer risk [13]. Combined with our study, we predicted that the T allele and T-carriers of rs6704688 were negatively correlated with the susceptibility to PsV from the additive model, whereas the heterozygous TC genotype had a protective effect from the heterozygous model. In the follow-up, we will conduct a detailed study of rs3834129.

Given the role of CASP7 in apoptosis and inflammation, the associations among CASP7 and autoimmune diseases and cancer susceptibility are important. Their relevance has been reported in insulin-dependent diabetes mellitus, rheumatoid arthritis, childhood leukemia, and different types of human cancers [18, 21]. Soung et al. found that a* CASP7* gene inactivating mutation (70 Cys to Tyr mutant) could lead to loss of apoptotic function [18]. Thus, we hypothesized that genetic variations in* CASP7* may act as strong apoptosis signals to block or delay the apoptosis of keratinocyte cells in PsV. The frequency of the haplotype GC in cases was lower than controls, which implied a protective role with regard to cases versus controls.

This study had some limitations. First, it is a hospital-based case-control study and the sample size was relatively small, which may limit the statistical power to explore the real association. In the future, we need to further study the larger sample size and ethnicity to validate our results. Next, we tested limited polymorphisms that were representative in* CASP *genes; these polymorphisms may not be the critical loci for psoriasis but may instead be nearby in LD with a causative locus. Studies on the correlations of more gene loci are needed. Additionally, more comprehensive studies, such as additional clinical studies and functional analyses, are needed to explain and supplement our findings. Despite these limitations, our study demonstrated the association between* CASP7* and* CASP8* polymorphisms and the PsV risk.

## 5. Conclusions

In conclusion, our findings demonstrated that* CASP7* and* CASP8* gene polymorphisms were associated with PsV risk in Han population of northeastern China. This is the first study describing the relationships between genetic polymorphisms in* CASP* family genes and PsV susceptibility in Han population in northeast China.* CASP7* and* CASP8* gene polymorphisms may be involved in the etiopathogenesis of PsV, and analysis of these polymorphisms could provide new insights into the diagnosis and treatment of PsV.

## Figures and Tables

**Table 1:** Characteristics of the study participants.

Characteristic	cases	controls	P value
Total Number	540	612	
Gender, n (%)			0.062
Male	332 (0.615)	342 (0.559)	
Female	208 (0.385)	270 (0.441)	
Age at onset, n (%)			
≤40 years	439 (0.813)		
>40 years	101 (0.187)		
Mean age ± SD, years	44.23±12.44	45.47±12.73	0.238
PASI, n (%)			
≤10	446 (0.826)		
>10	94 (0.174)		
Familial cases (N)	203 (0.376)		
Sporadic cases(N)	337 (0.624)		

N: number; PASI: psoriasis area and severity index.

**Table 2:** Candidate SNPs analysis.

Gene	SNP	Chromosome position	Major/minor allele	Risk allele	Risk allele frequency
					Case, Control
*CASP1*	rs2282659	11:105026710	A/G	A	0.804, 0.791
*CASP3*	rs2705897	4:184631944	T/G	G	0.193, 0.163
	rs4647610	4:184646777	T/C	C	0.359, 0.328
*CASP4*	rs547584	11:104943160	T/C	C	0.178, 0.157
	rs672016	11:104961127	C/G	G	0.228, 0.222
*CASP5*	rs507879	11:105007200	T/C	T	0.822, 0.804
*CASP6*	rs5030545	4:109699714	C/T	C	0.917, 0.913
*CASP7*	rs17090911	10:113719221	G/A	A	0.094, 0.072
	rs2227310	10:113729393	C/G	G	0.433, 0.384
*CASP8*	rs6704688	2:201241309	C/T	C	0.769, 0.753
	rs2293554	2:201266864	T/G	T	0.780, 0.758
*CASP9*	rs4233532	1:15495090	T/C	C	0.416, 0.413
	rs1052576	1:15506048	C/T	T	0.369, 0.368
*CASP10*	rs12613347	2:201190589	C/T	T	0.357, 0.351
	rs13006529	2:201217736	T/A	T	0.785, 0.783
*CASP12*	rs506601	11:104899754	A/T	A	0.698, 0.685
*CASP14*	rs3181304	19:15050098	A/G	G	0.435, 0.413

SNP: single nucleotide polymorphism.

**Table 3:** The single SNP association studies result of CASPs in cases and controls.

Gene Symbol	Genotype	Cases (n=540)	Controls (n= 612)	H-W P Value	P Value	Adjusted P Value	Statistical Model	P	P'	OR (95%CI)
*CASP1*	rs2282659									
	GG	24(0.044)	30(0.049)				Additive	0.8650	0.8813	
	GA	164(0.304)	196(0.320)	0.7871	0.5945	0.6279	Dominant	0.7952	0.8528	1.083
	AA	352(0.652)	386(0.630)				Recessive	0.5977	0.6057	(0.812,1.444)
	G/A	212/868	256/968				heterozygous	0.6687	0.7222	
		0.196/0.804	0.209/0.791							
*CASP3*	rs2705897									
	TT	350(0.648)	430(0.703)				Additive	0.3765	0.3845	
	TG	172(0.319)	164(0.268)	0.8629	0.1953	0.2205	Dominant	0.1632	0.1833	1.221
	GG	18(0.033)	18(0.029)				Recessive	0.7874	0.8057	(0.902,1.653)
	T/G	872/208	1024/200				heterozygous	0.1832	0.2056	
		0.807/0.193	0.837/0.163							
	rs4647610									
	TT	222(0.411)	274(0.448)				Additive	0.5322	0.5412	
	TC	248(0.459)	274(0.448)	0.1773	0.2700	0.2857	Dominant	0.3758	0.4025	1.147
	CC	70(0.130)	64(0.105)				Recessive	0.3498	0.3586	(0.899,1.463)
	T/C	692/388	822/402				heterozygous	0.7812	0.7998	
		0.641/0.359	0.672/0.328							
*CASP4*	rs547584									
	TT	368(0.681)	434(0.709)				Additive	0.5413	0.5451	
	TC	152(0.282)	164(0.268)	0.886	0.3451	0.3884	Dominant	0.4714	0.5293	1.162
	CC	20(0.037)	14(0.023)				Recessive	0.3164	0.3403	(0.852,1.585)
	T/C	888/192	1032/192				heterozygous	0.7170	0.7835	
		0.822/0.178	0.843/0.157							
	rs672016									
	GG	20(0.037)	30(0.049)				Additive	0.5789	0.5968	
	GC	206(0.382)	212(0.346)	1	0.8180	0.8317	Dominant	0.4794	0.5470	0.969
	CC	314(0.581)	370(0.605)				Recessive	0.5734	0.6178	(0.734,1.278)
	G/C	246/834	272/952				heterozygous	0.3824	0.3873	
		0.228/0.772	0.222/0.778							
*CASP5*	rs507879									
	TT	366(0.678)	402(0.657)				Additive	0.6168	0.6265	
	TC	156(0.289)	180(0.294)	1	0.4362	0.4632	Dominant	0.5950	0.5992	0.886
	CC	18(0.033)	30(0.049)				Recessive	0.3439	0.4141	(0.658,1.194)
	T/C	888/192	984/240				heterozygous	0.8904	0.9258	
		0.822/0.178	0.804/0.196							
*CASP6*	rs5030545									
	TT	2(0.004)	0(0)				Additive	0.4295	0.6065	
	TC	86(0.159)	106(0.173)	0.886	0.8369	0.9111	Dominant	-	0.9998	1.043
	CC	452(0.837)	506(0.827)				Recessive	0.7430	0.8257	(0.688,1.580)
	T/C	90/990	106/1118				heterozygous	0.6538	0.6495	
		0.083/0.917	0.087/0.913							
*CASP7*	rs17090911									
	GG	442(0.819)	526(0.860)				Additive	0.36448	0.4247	
	GA	94(0.174)	84(0.137)	0.8727	0.1609	0.1908	Dominant	0.1810	0.2086	1.346
	AA	4(0.007)	2(0.003)				Recessive	0.4888	0.6035	(0.884,2.051)
	G/A	978/102	1136/88				heterozygous	0.2229	0.2461	
		0.06/0.094	0.928/0.072							
	rs2227310									
	GG	104(0.193)	88(0.144)				Additive	0.2164	0.2147	
	GC	260(0.481)	294(0.480)	0.5244	0.0894	0.0919	Dominant	0.1172	0.1432	0.815
	CC	176(0.326)	230(0.376)				Recessive	0.2106	0.2184	(0.644,1.032)
	G/C	468/612	470/754				heterozygous	0.9792	0.9998	
		0.433/0.567	0.384/0.616							
*CASP8*	rs6704688									
	TT	40(0.074)	24(0.039)				Additive	0.0169*∗*	0.0179*∗*	
	TC	170(0.315)	254(0.415)	0.8725	0.5429	0.5777	Dominant	0.0679	0.0974	1.088
	CC	330(0.611)	334(0.546)				Recessive	0.1129	0.1258	(0.829,1.427)
	T/C	250/830	302/922				heterozygous	0.0126*∗*	0.0149*∗*	
		0.231/0.769	0.247/0.753							
	rs2293554									
	TT	332(0.615)	358(0.585)				Additive	0.6952	0.6784	
	TG	178(0.329)	212(0.346)	0.546	0.3999	0.4151	Dominant	0.4656	0.4911	0.886
	GG	30(0.056)	42(0.069)				Recessive	0.5166	0.6077	(0.673,1.167)
	T/G	842/238	928/296				heterozygous	0.6711	0.7163	
		0.780/0.220	0.758/0.242							
*CASP9*	rs4233532									
	TT	176(0.326)	224(0.366)				Additive	0.2005	0.2144	
	TC	278(0.515)	270(0.441)	0.0447	0.9114	0.9544	Dominant	0.3129	0.3380	1.013
	CC	86(0.159)	118(0.193)				Recessive	0.2915	0.3272	(0.801,1.281)
	T/C	630/450	718/506				heterozygous	0.0773	0.0821	
		0.584/0.416	0.587/0.413							
	rs1052576									
	TT	70(0.130)	98(0.160)				Additive	0.2790	0.2851	
	TC	258(0.478)	254(0.415)	1	0.9761	0.9999	Dominant	0.2994	0.3467	0.996
	CC	212(0.392)	260(0.425)				Recessive	0.4321	0.4519	(0.784,1.266)
	T/C	398/682	450/774				heterozygous	0.1304	0.1623	
		0.369/0.631	0.368/0.632							
*CASP10*	rs12613347									
	TT	74(0.137)	82(0.134)				Additive	0.9752	0.9714	
	TC	238(0.441)	266(0.435)	0.6406	0.8325	0.8550	Dominant	0.9150	0.9998	0.974
	CC	228(0.422)	264(0.431)				Recessive	0.8247	0.8681	(0.764,1.240)
	T/C	386/694	430/794				heterozygous	0.8829	0.9316	
		0.357/0.643	0.351/0.649							
	rs13006529									
	TT	342(0.633)	366(0.598)				Additive	0.0846	0.0806	
	TA	164(0.304)	226(0.369)	0.4431	0.9179	0.9452	Dominant	0.3849	0.3863	0.985
	AA	34(0.063)	20(0.033)				Recessive	0.0856	0.1072	(0.744,1.305)
	T/A	848/232	958/266				heterozygous	0.0965	0.1112	
		0.785/0.215	0.783/0.217							
*CASP12*	rs506601									
	TT	48(0.089)	48(0.078)				Additive	0.5086	0.5049	
	TA	230(0.426)	290(0.484)	0.6716	0.6101	0.6427	Dominant	0.6507	0.7603	1.065
	AA	262(0.485)	274(0.448)				Recessive	0.3682	0.4018	(0.829,1.369)
	T/A	326/754	386/838				heterozygous	0.2485	0.2688	
		0.302/0.698	0.315/0.685							
*CASP14*	rs3181304									
	GG	120(0.222)	104(0.170)				Additive	0.2007	0.2129	
	GA	230(0.426)	298(0.487)	1	0.4686	0.4826	Dominant	0.1139	0.1425	0.915
	AA	190(0.352)	210(0.343)				Recessive	0.8265	0.8582	(0.724,1.156)
	G/A	470/610	506/718				heterozygous	0.1424	0.1547	
		0.435/0.565	0.413/0.587							

*∗*: Significant, compared by paired t test; H-W: Hardy–Weinberg equilibrium test; P: model-based statistical p value; P': p value adjusted by permutation; OR: odds ratio; and 95%CI: 95% confidence interval.

**Table 4:** Significant results of intergenic interaction analysis of all samples.

SNP (Chr.)	SNP (Chr.)	OR_INT	Chi-square	P
rs12613347(2)	rs672016(11)	1.87	6.8995	0.0086
rs13006529(2)	rs506601(11)	0.384	6.6773	0.0098
rs13006529(2)	rs4647610(4)	3.039	6.0643	0.0138
rs4233532(1)	rs13006529(2)	0.430	4.9705	0.0258
rs6704688(2)	rs4647610(4)	0.54	4.6476	0.0311
rs12613347(2)	rs2282659(11)	0.41	4.6218	0.0316
rs506601(11)	rs2282659(11)	0.43	4.5832	0.0323
rs6704688(2)	rs672016(11)	0.58	4.5381	0.0332

SNP: single nucleotide polymorphism; Chr.: chromosome of single nucleotide polymorphism; OR_INT: odds ratio for interaction; P: p-value.

**Table 5:** Significant results of intergene interaction analysis of samples less than 40 years old.

SNP (Chr.)	SNP (Chr.)	OR_INT	Chi-square	P
rs13006529(2)	rs6704688(2)	4.85	8.9000	0.0029
rs5030545(4)	rs2705897(4)	0.12	6.1767	0.0129
rs4647610(4)	rs547584(11)	2.90	5.9144	0.0150
rs2293554(2)	rs4647610(4)	2.45	5.2208	0.0223
rs4233532(1)	rs1052576(1)	0.50	4.9522	0.0261
rs5030545(4)	rs4647610(4)	0.24	4.6047	0.0319
rs6704688(2)	rs2282659(11)	0.34	4.2747	0.0387
rs5030545(4)	rs17090911(10)	0.08	4.0495	0.0442
rs4233532(1)	rs2227310(10)	1.82	3.9662	0.0464
rs13006529(2)	rs2282659(11)	0.34	3.9280	0.0475

SNP: single nucleotide polymorphism; Chr.: chromosome of single nucleotide polymorphism; OR_INT: odds ratio for interaction; P: p-value.

**Table 6:** The haplotype analyses result of CASPs in cases and controls.

Gene	Haplotype	Freq.	Case, Control Ratio Counts	Case, Control Frequencies	Chi Square	P Value
CASP3	TT	0.646	341.8 : 198.2, 401.9 : 210.1	0.633, 0.657	0.712	0.3989
	TC	0.177	94.2 : 445.8, 110.1 : 501.9	0.174, 0.180	0.057	0.8118
	GC	0.166	99.8 : 440.2, 90.9 : 521.1	0.185, 0.149	2.721	0.099
	GT	0.012	4.2 : 535.8, 9.1 : 602.9	0.008, 0.015	1.234	0.2666
CASP4	TC	0.632	334.5 : 205.5, 393.5 : 218.5	0.619, 0.643	0.685	0.408
	TG	0.201	109.5 : 430.5, 122.5 : 489.5	0.203, 0.200	0.013	0.911
	CC	0.143	82.5 : 457.5, 82.5 : 529.5	0.153, 0.135	0.758	0.384
	CG	0.023	13.5 : 526.5, 13.5 : 598.5	0.025, 0.022	0.106	0.7446
CASP7	GC	0.51	255.0 : 285.0, 333.0 : 279.0	0.472, 0.544	5.934	0.0149^*∗*^
	GG	0.407	234.0 : 306.0, 235.0 : 377.0	0.433, 0.384	2.894	0.0889
	AC	0.082	51.0 : 489.0, 44.0 : 568.0	0.094, 0.072	1.928	0.165
CASP8	CT	0.546	304.0 : 236.0, 325.2 : 286.8	0.563, 0.531	1.157	0.282
	TT	0.222	117.0 : 423.0, 138.8 : 473.2	0.217, 0.227	0.172	0.6787
	CG	0.214	111.0 : 429.0, 135.8 : 476.2	0.205, 0.222	0.457	0.4991
	TG	0.018	8.0 : 532.0, 12.2 : 599.8	0.015, 0.020	0.43	0.5121
CASP9	TC	0.582	314.9 : 225.1, 355.9 : 256.1	0.583, 0.582	0.003	0.9559
	CT	0.365	198.9 : 341.1, 221.9 : 390.1	0.368, 0.363	0.041	0.8397
	CC	0.05	26.1 : 513.9, 31.1 : 580.9	0.048, 0.051	0.037	0.8465
CASP10	CT	0.431	232.3 : 307.7, 264.4 : 347.6	0.430, 0.432	0.004	0.9506
	TT	0.353	191.7 : 348.3, 214.6 : 397.4	0.355, 0.351	0.023	0.8783
	CA	0.215	114.7 : 425.3, 132.6 : 479.4	0.212, 0.217	0.031	0.8596

*∗*: significant; Freq.: frequencies.

**Table 7:** Haplotype analysis of SNPs on the same chromosome for all samples.

Chr.	SNP	Haplotype	Ca-freq.	N-Ca (540)	Co-freq.	N-Co ( 612)	P
1	rs4233532	CC	0.048	25.92	0.053	32.436	0.9226
	rs1052576	CT	0.369	199.26	0.364	222.768	
		TC	0.583	314.82	0.583	356.796	
11	rs506601	ACCCG	0.034	18.36	0.027	16.524	0.0093
	rs547584	ACCTA	0.116	62.64	0.102	62.424	
	rs672016	ACGTA	0.028	15.12	0.023	14.076	
	rs507879	ATCCG	0.057	30.78	0.067	41.004	
	rs2282659	ATCTA	0.265	143.1	0.277	169.524	
		ATCTG	0.014	7.56	0.022	13.464	
		ATGCG	0.049	26.46	0.041	25.092	
		ATGTA	0.141	76.14	0.139	85.068	
		TTCCG	0.033	17.82	0.047	28.764	
		TTCTA	0.245	132.3	0.238	145.656	
		TTCTG	0.018	9.72	0	0	
		TTGTA	0	0	0.017	10.404	
10	rs17090911	AC	0.094	50.76	0.072	44.064	0.0448
	rs2227310	GC	0.473	255.42	0.544	332.928	
		GG	0.433	233.82	0.384	235.008	
2	rs12613347	CACT	0	0	0.012	7.344	0.0038
	rs13006529	CATG	0	0	0.01	6.12	
	rs6704688	CATT	0.2	108	0.196	119.952	
	rs2293554	CTCG	0.159	85.86	0.164	100.368	
		CTCT	0.263	142.02	0.248	151.776	
		CTTT	0.023	12.42	0.023	14.076	
		TTCG	0.05	27	0.064	39.168	
		TTCT	0.305	164.7	0.268	164.016	
		TTTT	0	0	0.015	9.18	
4	rs2705897	CGC	0.179	96.66	0.124	75.888	0.00004
	rs4647610	CGT	0	0	0.016	9.792	
	rs5030545	CTC	0.164	88.56	0.161	98.532	
		CTT	0.576	311.04	0.613	375.156	
		TGC	0	0	0.023	14.076	
		TTC	0.017	9.18	0.02	12.24	
		TTT	0.064	34.56	0.043	26.316	

Chr.: chromosome; SNP: single nucleotide polymorphism; N-Ca: number of case group; Ca-freq.: case group frequencies; N-Co: number of control group; Co-freq.: control group frequencies; P: p value.

**Table 8:** Haplotype analysis of SNPs on the same chromosome for samples under 40 years old.

chr	SNP	Haplotype	Ca-freq	N-Ca ( 439)	Co-freq	N-Co (216)	P
1	rs4233532	CC	0.545	239.255	0.051	11.016	0.00000
	rs1052576	CT	0.401	176.039	0.338	73.008	
		TC	0.054	23.706	0.611	131.976	
11	rs506601	ACCCG	0.042	18.438	0.019	4.104	0.00098
	rs547584	ACCTA	0.119	52.241	0.105	22.68	
	rs672016	ACGTA	0.035	15.365	0.024	5.184	
		ATCCA	0	0	0.019	4.104	
	rs507879	ATCCG	0.027	11.853	0.058	12.528	
	rs2282659	ATCTA	0.309	135.651	0.224	48.384	
		ATCTG	0.015	6.585	0.038	8.208	
		ATGCG	0.053	23.267	0.043	9.288	
		ATGTA	0.117	51.363	0.172	37.152	
		TTCCG	0.026	11.414	0.054	11.664	
		TTCTA	0.227	99.653	0.229	49.464	
		TTCTG	0.018	7.902	0	0	
		TTGTA	0.012	5.268	0.015	3.24	
10	rs17090911	AC	0.094	41.266	0.102	22.032	0.08798
	rs2227310	GC	0.436	191.404	0.518	111.888	
		GG	0.47	206.33	0.38	82.08	
2	rs12613347	CACT	0	0	0.015	3.24	0.00000
	rs13006529	CATT	0.176	77.264	0.187	40.392	
	rs6704688	CTCC	0	0	0.161	34.776	
	rs2293554	CTCG	0.198	86.922	0	0	
		CTCT	0.289	126.871	0.294	63.504	
		CTTT	0	0	0.028	6.048	
		TTCC	0	0	0.073	15.768	
		TTCG	0.075	32.925	0	0	
		TTCT	0.262	115.018	0.226	48.816	
		TTTT	0	0	0.016	3.456	
4	rs2705897	CGC	0.176	77.264	0.107	23.112	0.00001
	rs4647610	CGT	0	0	0.011	2.376	
	rs5030545	CTC	0.183	80.337	0.182	39.312	
		CTT	0.533	233.987	0.644	139.104	
		TGC	0	0	0.02	4.32	
		TTC	0.014	6.146	0.016	3.456	
		TTT	0.094	41.266	0.02	4.32	

Chr, Chromosome; SNP, single nucleotide polymorphism; N-Ca, number of case group; Ca-freq, case group frequencies; N-Co, Number of control group; Co-freq, control group frequencies; P, p value.

## Data Availability

According to the informed consent signed by the volunteers, the aggregated SNP genotype frequency and allele frequency data in the manuscript can be available freely by the academic community. The SNP genotype and the clinical information can be claimed by e-mail after security audit.

## Supplementary Materials

Supplementary Materials The supplementary materials contain several experimental data and experimental results that are closely related to the content of the manuscript. They are relatively unimportant or large or negative results, but they are still very meaningful for manuscripts.
