# Supplementary material for: Association Study of the Caspase Gene Family and Psoriasis Vulgaris Susceptibility in Northeastern China
Source: Biomed Res Int. 2019 Feb 17;2019:2417612. doi: 10.1155/2019/2417612 (PMC6398065; doi:10.1155/2019/2417612)
Supplement: Supplementary Materials — The supplementary materials contain several experimental data and experimental results that are closely related to the content of the manuscript. They are relatively unimportant or large or negative results, but they are still very meaningful for manuscripts. [file 2417612.f1.zip › 2417612.f1/PDF file of the Supplementary material_BMRI_2661541.pdf]

Table S1 Genotyping primers.

Gene	SNP	PCR primer (5’-3’)	PCR primer (3’-5’)	Gene
CASP1	rs2282659	GCAGGAGCGGGGTGAAACTA	GACATCCCACAATGGGCTCTG	320
CASP3	rs2705897	CCATGGCTCAGAAGCACACAAA	CGTGCCCCAGGTTAGGTTAAGA	192
	rs4647610	TGGAGGATCTCGGATGCCTTTT	TGAGCATCGGTATAAGCCCTGAA	299
CASP4	rs547584	TCCATCTGTGACATCTTAGTCTATTCACCA	AAGCCAGATCGCGTGGTTCTATG	167
	rs672016	GGACAGGGGCCATGAACTACA	TGCACATTGGGTTGGCAGTG	362
CASP5	rs507879	CTGCATGGGCCTTGGAGTTG	GCAAAACACGATGTTCTGACATTGA	242
CASP6	rs5030545	CCTCCTGGAACTGCCCCAAG	TGTGCTCACCTAGGGCAAAGG	313
CASP7	rs17090911	TGCCACTGCAACTCCCATCTAA	AGGGTGACGCTGGGTTTTCTTC	228
	rs2227310	CCTGGTTTGTGCAAGCCCTCT	TTGGTGAGCATGGAGACCACAC	175
CASP8	rs6704688	TCGTGCCTCAAGGAGAGGAGAA	TGATTCAGTCATCGTAGATTGGAAGG	387
	rs2293554	GGGTTGAATGGACAGCCTCTGA	TTCCCAAAGCCTCCCAAGTGAT	230
CASP9	rs4233532	TCAGGCAGAGGAGAGGCAAGTG	GCCCACACCCAGTGACATCTTT	285
	rs1052576	TGGCTCCCAAGAAAACAACAGG	CCTATCCGTGCTTCTGGCTCAC	289
CASP10	rs12613347	TTTCAGTGCCCACATTTGCAGT	ACCCGCCACCTCATATTGTTCA	265
	rs13006529	TCGAAGAGTGGACAAACAGGGAAC	AGGGTGACGCTGGGTTTTCTTC	172
CASP12	rs506601	TCCCAGCCTTGTCAGCAGAGAT	TGTGGGACCAAAGATGAAAAGCATA	439
CASP14	rs3181304	GAGCTGCCAGTTCAGCCATGAG	CACACACACAGCAAGTGCTGTGA	346

Table S2 iMLDR probe sequences.

Gene	Snp Allele	Primer (5'-3')	LDR product
CASP1	rs2282659_modify:	CTCCAAAACTCTTTTGGAAAGAAGAGCTTTTTTTTTTTTTTTTTTTTT	64.78
	rs2282659_A:	TACGGTTATTCGGGCTCCTGTCTGGGTTTGTCCACTCTCCAAACAA	66.69
	rs2282659_G:	TTCCGCGTTCGGACTGATATCTGGGTTTGTCCACTCTCCAAACAG	66.41
CASP3	rs2705897_modify:	AAAGACATTTGCAAATAAAAGGAAAAAAATTTTTTTTTTTTTTTTTTTTTT	63.19
	rs2705897_G:	TCTCTCGGGTCAATTCGTCCTTTCCTTTTGCTGTGATCTTCTTTAGAAACAGG	68.02
	rs2705897_T:	TGTTCGTGGGCCGGATTAGTTCCTTTTGCTGTGATCTTCTTTAGAAACAGT	66.22
CASP3	rs4647610_modify:	AGTAGCATTTATTCATTTGATATTTGCACCTTTTTTTTTTTTTTTTTTTTTTTTT	63.47
	rs4647610_C:	TTCCGCGTTCGGACTGATATGGAAGACTGAAACTGCCAAAACTCAGAGTC	68.69
	rs4647610FT:	TACGGTTATTCGGGCTCCTGTGGAAGACTGAAACTGCCAAAACTCAGAGTT	68.18
CASP4	rs547584_modify:	TAATATGCTTTAAATTAGGTTTTACAAAGATCACATTTTTTTTTTTTTTTTTTTTT	63.20
	rs547584_C:	TCTCTCGGGTCAATTCGTCCTTGGAAGCCTTTCTAGTGCTTTAGAAAAAAGTAG	66.24
	rs547584_T:	TGTTCGTGGGCCGGATTAGTGGAAGCCTTTCTAGTGCTTTAGAAAAAAGCAA	66.45
CASP4	rs672016_modify:	TAGCCCAAGTAGCCGCCTAGTTTTTTTTTTTT	63.44
	rs672016_C:	TACGGTTATTCGGGCTCCTGTTCCAGCGATGGATCCAGATGC	67.02
	rs672016_G:	TTCCGCGTTCGGACTGATATTCCAGCGATGGATCCAGATGG	66.87
CASP5	rs507879_modify:	ATCATAATATTTTTTCTTTTCCTCTTCCTTCATTTTTTTTTTTTTTTTTTTTTTTTT	63.53
	rs507879_C:	TTCCGCGTTCGGACTGATATCAGGGCCTTGTCTTCAATTTTTGC	68.70
	rs507879_T:	TACGGTTATTCGGGCTCCTGTCAGGGCCTTGTCTTCAATTTTCGT	66.44
CASP6	rs5030545_modify:	GGGAGGACCAGTTGGAGAGGTTTTTTT	63.30
	rs5030545_C:	TTCCGCGTTCGGACTGATATGCTTTCTCATCTGCCATGCATCG	70.38
	rs5030545_T:	TACGGTTATTCGGGCTCCTGTGCTTTCTCATCTGCCATGCACCA	69.00
CASP7	rs17090911_modify:	CTGATCYGGTGAGTTGTTACCCTTTTTTTT	63.64
	rs17090911_A:	TGTTCGTGGGCCGGATTAGTCGTCCACCYGCCCGTGTCAT	72.85
	rs17090911_G:	TCTCTCGGGTCAATTCGTCCTTCGTCCACCYGCCCGTGTCAC	73.59
CASP7	rs2227310_modify:	CTGGAAATCATGCARATCCTCACTTTTTTTTTTT	63.13
	rs2227310_C:	TACGGTTATTCGGGCTCCTGTCCTGGAGGAGCACGGAAAACAC	66.54
	rs2227310_G:	TTCCGCGTTCGGACTGATATCCTGGAGGAGCACGGAAAACAG	66.61
CASP8	rs6704688_modify:	TAGTTAGGCTAAGTAAGAAAGAGAGAAGACTCAAATTTTTTTTTTTTTTTTTTTTTT	63.69
	rs6704688_C:	TTCCGCGTTCGGACTGATATGTTTTTTGAAAAGATAAACAAAATCAACAAATACC	67.81
	rs6704688_T:	TACGGTTATTCGGGCTCCTGTGTTTTTTGAAAAGATAAACAAAATCAACAAATGCT	
CASP8	rs2293554_modify:	AAGCYCCAATCAGCTCAGAGGTTTTTTTTTTTTTT	65.10
	rs2293554_G:	TTCCGCGTTCGGACTGATATGGCACTAGGCAGGGTACCACAAACAC	68.42
	rs2293554_T:	TACGGTTATTCGGGCTCCTGTGGCACTAGGCAGGGTACCACAAACAA	68.71
CASP9	rs4233532_modify:	GCGCATCCTTGCCCGCTCAGTTTTTTT	72.65
	rs4233532_C:	TTCCGCGTTCGGACTGATATGGCGCAGGCCTCCTTGCAGC	3.77

	rs4233532_T:	TACGGTTATTCGGGCTCCTGTGGCGCAGGCCTCCTTGCTGT	71.33
CASP9	rs1052576_modify:	GCAGGACCACGGTGCTCTGGTTTTTTT	70.08
	rs1052576_T:	TGTTCGTGGGCCGGATTAGTGCTTTGCTGGAGCTGGCTCA	68.80
	rs1052576_C:	TCTCTCGGGTCAATTCGTCCTTGCTTTGCTGGAGCTGGCACG	66.35
CASP10	rs12613347_modify:	GCTCACAAATTTTCCAGTGTCTAGGTTTTTTTTTTTTTTTTTTTT	63.04
	rs12613347_C:	TTCCGCGTTCGGACTGATATTCTCTCCCAAGTTGGGGCAACC	68.77
	rs12613347_T:	TACGGTTATTCGGGCTCCTGTTCTCTCCCAAGTTGGGGCAGCT	66.45
CASP10	rs13006529_modify:	TATAGCAGAGAGTTTTTGTTGGTTCTTAGACCTTTTTTTTTTTTTTTTTTTT	64.92
	rs13006529_A:	TCTCTCGGGTCAATTCGTCCTTTGCCCCTGGATGCACTTTGAA	68.12
	rs13006529_T:	TGTTCGTGGGCCGGATTAGTTGCCCCTGGATGCACTTTGAT	67.10
CASP12	rs506601_modify:	CTTCACAATGTGTGACAAACATTTTAAAATTTTTTTTTTTTTTTTTTTTTTT	63.25
	rs506601_A:	TTCCGCGTTCGGACTGATATCTTAAAATTATATTATTGATAATTTGCTTTATTGA A	58.43
	rs506601_T:	TACGGTTATTCGGGCTCCTGTCTTAAAATTATATTATTGATAATTTGCTTTATTG AT	57.85
CASP14	rs3181304_modify:	CCATTGCTGTTAAGCACCTAGAGCTTTTTTTTTTTTTTTTTTTTT	63.47
	rs3181304_A:	TACGGTTATTCGGGCTCCTGTGGGATCCTAAAACCTACCTGGGACA	66.27
	rs3181304_G:	TTCCGCGTTCGGACTGATATGGGATCCTAAAACCTACCTGGGACG	67.56

Table S3. Non-significant results of intergenic interaction analysis of all samples

SNP(Chr)	SNP(Chr)	OR_INT	Chi-square	P
rs12613347(2)	rs672016(11)	1.87088	6.89948	0.008622
rs13006529(2)	rs506601(11)	0.380435	6.67732	0.009765
rs13006529(2)	rs4647610(4)	3.02893	6.06429	0.01379
rs4233532(1)	rs13006529(2)	0.433298	4.97048	0.02578
rs6704688(2)	rs4647610(4)	0.54489	4.64764	0.0311
rs12613347(2)	rs2282659(11)	0.413222	4.62179	0.03157
rs506601(11)	rs2282659(11)	0.433815	4.58316	0.03229
rs6704688(2)	rs672016(11)	0.579687	4.53809	0.03315
rs13006529(2)	rs2227310(10)	1.90046	3.58744	0.05822
rs2227310(10)	rs2282659(11)	0.54177	3.31672	0.06858
rs4647610(4)	rs672016(11)	0.632569	3.03089	0.08169
rs17090911(10)	rs2282659(11)	0.589216	2.83441	0.09226
rs13006529(2)	rs17090911(10)	1.67234	2.76846	0.09614
rs4647610(4)	rs2282659(11)	0.520418	2.57828	0.1083
rs13006529(2)	rs2282659(4)	0.307957	2.41556	0.1201
rs2227310(10)	rs547584(11)	1.44416	2.39628	0.1216
rs12613347(2)	rs6704688(2)	0.676949	2.25926	0.1328
rs506601(11)	rs507879(11)	0.695608	2.20244	0.1378
rs506601(11)	rs547584(11)	0.639831	2.09245	0.148
rs672016(11)	rs507879(11)	0.691495	2.03727	0.1535
rs1052576(1)	rs17090911(10)	0.771038	2.01856	0.1554
rs672016(11)	rs2282659(11)	0.61121	1.91344	0.1666
rs4233532(1)	rs2227310(10)	1.28171	1.84207	0.1747
rs5030545(4)	rs547584(11)	0.730925	1.82892	0.1763
rs4233532(1)	rs2705897(4)	0.763966	1.68777	0.1939
rs4233532(1)	rs4647610(4)	1.33038	1.5287	0.2163
rs4233532(1)	rs672016(11)	0.742534	1.51538	0.2183
rs2293554(2)	rs3181304(19)	0.77683	1.49299	0.2218
rs1052576(1)	rs13006529(2)	1.45051	1.4818	0.2235
rs12613347(2)	rs506601(11)	0.756185	1.47068	0.2252
rs13006529(2)	rs547584(11)	1.72357	1.46928	0.2255
rs12613347(4)	rs4647610(4)	0.725222	1.46773	0.2257
rs4647610(4)	rs507879(11)	0.710117	1.3907	0.2383
rs1052576(1)	rs6704688(2)	1.26616	1.37666	0.2407
rs6704688(2)	rs17090911(10)	1.26762	1.3755	0.2409
rs1052576(1)	rs2282659(11)	0.695662	1.36046	0.2435
rs507879(11)	rs3181304(19)	1.33232	1.24832	0.2639
rs6704688(2)	rs2282659(11)	0.639381	1.23578	0.2663
rs5030545(4)	rs2282659(11)	1.36171	1.11458	0.2911
rs2227310(10)	rs672016(11)	0.805169	1.10649	0.2928
rs13006529(2)	rs2705897(4)	1.4222	0.948353	0.3301
rs13006529(2)	rs2293554(2)	1.52215	0.946049	0.3307

rs1052576(1)	rs507879(11)	1.2219	0.939305	0.3325
rs2227310(10)	rs506601(11)	0.826337	0.937518	0.3329
rs2293554(2)	rs5030545(4)	1.1738	0.934169	0.3338
rs4233532(1)	rs506601(11)	1.20323	0.925688	0.336
rs12613347(2)	rs547584(11)	0.780187	0.92355	0.3365
rs506601(11)	rs3181304(19)	0.783453	0.884717	0.3469
rs6704688(2)	rs2227310(10)	0.811948	0.84905	0.3568
rs5030545(4)	rs4647610(4)	1.22329	0.820584	0.365
rs2293554(2)	rs547584(11)	1.22551	0.779388	0.3773
rs507879(11)	rs2282659(11)	0.699858	0.764556	0.3819
rs17090911(10)	rs507879(11)	1.19558	0.755069	0.3849
rs2705897(4)	rs4647610(4)	1.25383	0.722399	0.3954
rs2293554(2)	rs2282659(11)	1.29398	0.714354	0.398
rs2705897(4)	rs547584(11)	0.797241	0.691812	0.4055
rs17090911(10)	rs547584(11)	1.22151	0.691342	0.4057
rs6704688(2)	rs2705897(4)	0.809578	0.671583	0.4125
rs6704688(2)	rs5030545(4)	0.840171	0.66854	0.4136
rs547584(11)	rs672016(11)	0.814932	0.667406	0.414
rs12613347(2)	rs507879(11)	0.810081	0.657286	0.4175
rs4233532(1)	rs6704688(2)	1.1792	0.636437	0.425
rs2293554(2)	rs672016(11)	1.17678	0.615379	0.4328
rs6704688(2)	rs547584(11)	0.813053	0.577134	0.4474
rs506601(11)	rs672016(11)	0.840128	0.576201	0.4478
rs2227310(10)	rs3181304(19)	0.84311	0.574135	0.4486
rs6704688(2)	rs3181304(19)	1.20849	0.565634	0.452
rs1052576(1)	rs547584(11)	1.18922	0.562924	0.4531
rs547584(11)	rs2282659(11)	0.758866	0.498677	0.4801
rs2705897(4)	rs672016(11)	0.825742	0.492009	0.483
rs1052576(1)	rs2705897(4)	1.14044	0.490512	0.4837
rs4233532(1)	rs507879(11)	1.15808	0.464579	0.4955
rs12613347(2)	rs3181304(19)	1.17661	0.455737	0.4996
rs2705897(4)	rs2227310(10)	1.15699	0.449123	0.5028
rs17090911(10)	rs672016(11)	0.873979	0.445031	0.5047
rs2293554(2)	rs506601(11)	1.13376	0.412371	0.5208
rs1052576(1)	rs672016(11)	0.875703	0.391196	0.5317
rs5030545(4)	rs507879(11)	0.880494	0.353504	0.5521
rs1052576(1)	rs2227310(10)	1.1122	0.336675	0.5618
rs2282659(11)	rs3181304(19)	1.26445	0.328156	0.5667
rs13006529(2)	rs3181304(19)	1.24908	0.323238	0.5697
rs5030545(4)	rs506601(11)	0.901631	0.317042	0.5734
rs5030545(4)	rs2227310(10)	0.909314	0.306921	0.5796
rs13006529(2)	rs6704688(2)	0.802571	0.304457	0.5811
rs2705897(4)	rs507879(11)	0.869665	0.277022	0.5987
rs2705897(4)	rs2282659(11)	1.19926	0.262991	0.6081

rs6704688(2)	rs2293554(2)	0.900894	0.248531	0.6181
rs5030545(4)	rs672016(11)	0.909785	0.242418	0.6225
rs13006529(2)	rs5030545(4)	0.858009	0.229101	0.6322
rs672016(11)	rs3181304(19)	1.11736	0.219557	0.6394
rs4647610(4)	rs17090911(10)	0.907477	0.209144	0.6474
rs6704688(2)	rs506601(11)	0.899519	0.208401	0.648
rs2293554(2)	rs507879(11)	0.910629	0.198247	0.6561
rs4233532(1)	rs3181304(19)	1.09429	0.192432	0.6609
rs13006529(2)	rs672016(11)	1.18713	0.192105	0.6612
rs4233532(1)	rs12613347(2)	0.890036	0.174265	0.6763
rs2705897(4)	rs3181304(19)	0.904446	0.167317	0.6825
rs6704688(2)	rs507879(11)	0.899691	0.154929	0.6939
rs1052576(1)	rs2293554(2)	1.06852	0.152535	0.6961
rs12613347(2)	rs13006529(2)	1.1629	0.141733	0.7066
rs2293554(2)	rs4647610(4)	0.922943	0.140313	0.708
rs2705897(4)	rs17090911(10)	1.06805	0.132593	0.7158
rs4233532(1)	rs1052576(1)	1.06596	0.132095	0.7163
rs4233532(1)	rs547584(11)	0.919935	0.130972	0.7174
rs2293554(2)	rs17090911(10)	1.06189	0.130084	0.7183
rs1052576(1)	rs12613347(2)	0.926385	0.122169	0.7267
rs12613347(2)	rs2227310(10)	0.930578	0.119546	0.7295
rs547584(11)	rs3181304(19)	0.912289	0.105917	0.7448
rs4647610(4)	rs506601(11)	0.928054	0.086357	0.7689
rs1052576(1)	rs3181304(19)	1.05884	0.078827	0.7789
rs4233532(1)	rs5030545(4)	1.04713	0.076873	0.7816
rs13006529(2)	rs507879(11)	0.894779	0.07357	0.7862
rs1052576(1)	rs5030545(4)	1.04652	0.072894	0.7872
rs4647610(4)	rs547584(11)	0.92635	0.068305	0.7938
rs2293554(2)	rs2227310(10)	1.04372	0.056335	0.8124
rs2293554(2)	rs2705897(4)	0.956494	0.055674	0.8135
rs547584(11)	rs507879(11)	0.943973	0.043625	0.8346
rs12613347(2)	rs2293554	1.04787	0.04347	0.8348
rs4233532(1)	rs17090911	1.03513	0.038046	0.8454
rs4647610(4)	rs2227310	1.0509	0.03661	0.8483
rs5030545(4)	rs3181304(19)	1.03532	0.029558	0.8635
rs17090911(10)	rs2227310	0.972697	0.023296	0.8787
rs4647610(4)	rs3181304(19)	1.04422	0.022803	0.88
rs2227310(10)	rs507879(11)	1.03331	0.020342	0.8866
rs17090911(10)	rs3181304(19)	1.02568	0.016447	0.898
rs12613347(2)	rs17090911(10)	0.975234	0.014048	0.9057
rs2705897(4)	rs506601(11)	1.02578	0.013461	0.9076
rs5030545(4)	rs2705897(4)	1.02122	0.012416	0.9113
rs12613347(2)	rs2705897(4)	0.973098	0.008896	0.9249
rs12613347(2)	rs5030545(4)	1.01575	0.006178	0.9373

rs1052576(1)	rs506601(11)	0.985625	0.006125	0.9376
rs4233532(1)	rs2282659(11)	1.01487	0.001887	0.9653
rs1052576(1)	rs4647610(4)	0.993118	0.001114	0.9734
rs17090911(10)	rs506601(11)	1.00553	0.000881	0.9763
rs4233532(1)	rs2293554(2)	1.00453	0.000687	0.9791
rs5030545(4)	rs17090911(10)	0.997939	0.000149	0.9902

SNP, single nucleotide polymorphism; Chr, Chromosome of single nucleotide polymorphism; OR_INT, Odds ratio for interaction; P, p-value

Table S4. Non-significant results of inter-gene interaction analysis of samples less than 40 years old

SNP1	SNP2	OR_INT	Chi-square	P
rs4233532(1)	rs1052576(1)	0.500484	4.95215	0.02606
rs4233532	rs12613347(2)	0.73771	1.0474	0.3061
rs4233532	rs13006529(2)	1.56987	1.25533	0.2625
rs4233532	rs6704688(2)	1.29167	0.464027	0.4957
rs4233532	rs2293554(2)	0.879576	0.193231	0.6602
rs4233532	rs5030545(4)	0.732309	0.278148	0.5979
rs4233532	rs2705897(4)	0.754532	0.509995	0.4751
rs4233532	rs4647610(4)	1.14684	0.172763	0.6777
rs4233532	rs17090911(10)	1.91367	1.82988	0.1761
rs4233532	rs2227310(10)	1.81878	3.9662	0.04642
rs4233532	rs506601(11)	1.07334	0.048857	0.8251
rs4233532	rs547584(11)	1.26819	0.340463	0.5596
rs4233532	rs672016(11)	1.01436	0.001817	0.966
rs4233532	rs507879(11)	1.25217	0.408887	0.5225
rs4233532	rs2282659(11)	1.17881	0.238953	0.625
rs4233532	rs3181304(19)	1.13988	0.202368	0.6528
rs1052576(1)	rs12613347(2)	0.734111	1.1166	0.2907
rs1052576	rs13006529(2)	1.51724	1.00963	0.315
rs1052576	rs6704688(2)	1.28636	0.426095	0.5139
rs1052576	rs2293554(2)	0.824896	0.410874	0.5215
rs1052576	rs5030545(4)	0.882317	0.04303	0.8357
rs1052576	rs2705897(4)	0.940507	0.026431	0.8709
rs1052576	rs4647610(4)	1.2279	0.354878	0.5514
rs1052576	rs17090911(10)	1.49908	0.786605	0.3751
rs1052576	rs2227310(10)	1.74155	3.31136	0.0688
rs1052576	rs506601(11)	1.1119	0.107367	0.7432
rs1052576	rs547584(11)	1.18366	0.177944	0.6731
rs1052576	rs672016(11)	1.06869	0.036367	0.8488
rs1052576	rs507879(11)	1.22373	0.316181	0.5739
rs1052576	rs2282659(11)	1.1632	0.196556	0.6575
rs1052576	rs3181304(19)	0.956704	0.023143	0.8791
rs12613347(2)	rs13006529(2)	1.07681	0.018346	0.8923
rs12613347	rs6704688(2)	0.711423	0.558274	0.455
rs12613347	rs2293554(2)	0.714423	1.20181	0.273
rs12613347	rs5030545(4)	1.39516	0.27832	0.5978
rs12613347	rs2705897(4)	1.35253	0.501609	0.4788
rs12613347	rs4647610(4)	0.968516	0.010871	0.917
rs12613347	rs17090911(10)	0.43267	2.0747	0.1498
rs12613347	rs2227310(10)	0.979232	0.006084	0.9378
rs12613347	rs506601(11)	1.31798	0.69494	0.4045
rs12613347	rs547584(11)	0.748639	0.533255	0.4652
rs12613347	rs672016(11)	1.42123	1.1606	0.2813

rs12613347	rs507879(11)	1.57541	1.38588	0.2391
rs12613347	rs2282659(11)	1.47615	1.11482	0.291
rs12613347	rs3181304(19)	1.11002	0.131173	0.7172
rs13006529(2)	rs6704688(2)	4.84722	8.89996	0.002852
rs13006529	rs2293554(2)	0.879058	0.074297	0.7852
rs13006529	rs5030545(4)	0.534802	0.969065	0.3249
rs13006529	rs2705897(4)	0.419086	3.33315	0.0679
rs13006529	rs4647610(4)	0.59357	1.82545	0.1767
rs13006529	rs17090911(10)	0.431271	1.65213	0.1987
rs13006529	rs2227310(10)	1.34796	0.579206	0.4466
rs13006529	rs506601(11)	0.723868	0.590365	0.4423
rs13006529	rs547584(11)	0.906464	0.04475	0.8325
rs13006529	rs672016(11)	1.32243	0.422269	0.5158
rs13006529	rs507879(11)	0.341075	3.14966	0.07594
rs13006529	rs2282659(11)	0.34197	3.92804	0.04749
rs13006529	rs3181304(19)	0.829898	0.315742	0.5742
rs6704688(2)	rs2293554(2)	0.768402	0.325137	0.5685
rs6704688	rs5030545(4)	0.872099	0.073418	0.7864
rs6704688	rs2705897(4)	0.492185	2.2981	0.1295
rs6704688	rs4647610(4)	0.604051	1.76515	0.184
rs6704688	rs17090911(10)	0.687349	0.320075	0.5716
rs6704688	rs2227310(10)	1.62567	1.69793	0.1926
rs6704688	rs506601(11)	1.08835	0.043105	0.8355
rs6704688	rs547584(11)	1.08547	0.032909	0.856
rs6704688	rs672016(11)	0.836206	0.181155	0.6704
rs6704688	rs507879(11)	0.390537	2.52758	0.1119
rs6704688	rs2282659(11)	0.339471	4.27468	0.03868
rs6704688	rs3181304(19)	1.05008	0.022479	0.8808
rs2293554(2)	rs5030545(4)	0.947435	0.008905	0.9248
rs2293554	rs2705897(4)	1.53725	0.826111	0.3634
rs2293554	rs4647610(4)	2.45224	5.22077	0.02232
rs2293554	rs17090911(10)	1.75171	1.02167	0.3121
rs2293554	rs2227310(10)	0.653939	2.06846	0.1504
rs2293554	rs506601(11)	0.896776	0.105223	0.7456
rs2293554	rs547584(11)	0.746584	0.482849	0.4871
rs2293554	rs672016(11)	0.694509	0.979045	0.3224
rs2293554	rs507879(11)	1.14932	0.095373	0.7575
rs2293554	rs2282659(11)	0.952456	0.013004	0.9092
rs2293554	rs3181304(19)	1.19779	0.301538	0.5829
rs5030545(4)	rs2705897(4)	0.122696	6.1767	0.01294
rs5030545	rs4647610(4)	0.243778	4.60466	0.03189
rs5030545	rs17090911(10)	0.08234	4.04946	0.04419
rs5030545	rs2227310(10)	0.962213	0.0052	0.9425
rs5030545	rs506601(11)	0.39033	2.3118	0.1284

rs5030545	rs547584(11)	0.631523	0.524085	0.4691
rs5030545	rs672016(11)	1.19673	0.070024	0.7913
rs5030545	rs507879(11)	0.498419	0.98531	0.3209
rs5030545	rs2282659(11)	0.43848	1.2635	0.261
rs5030545	rs3181304(19)	1.6296	0.855262	0.3551
rs2705897(4)	rs4647610(4)	0.484373	1.98496	0.1589
rs2705897	rs17090911(10)	2.27607	1.35217	0.2449
rs2705897	rs2227310(10)	0.972657	0.005684	0.9399
rs2705897	rs506601(11)	0.476877	2.37964	0.1229
rs2705897	rs547584(11)	1.33447	0.291701	0.5891
rs2705897	rs672016(11)	1.48406	0.679833	0.4096
rs2705897	rs507879(11)	2.00798	1.65796	0.1979
rs2705897	rs2282659(11)	1.64458	0.871117	0.3506
rs2705897	rs3181304(19)	1.36572	0.584167	0.4447
rs4647610(4)	rs17090911(10)	1.42186	0.323734	0.5694
rs4647610	rs2227310(10)	0.816066	0.474276	0.491
rs4647610	rs506601(11)	0.905952	0.081215	0.7757
rs4647610	rs547584(11)	2.90082	5.91442	0.01502
rs4647610	rs672016(11)	0.720905	0.764754	0.3818
rs4647610	rs507879(11)	1.19126	0.185494	0.6667
rs4647610	rs2282659(11)	1.04906	0.012767	0.91
rs4647610	rs3181304(19)	0.72408	1.02716	0.3108
rs17090911(10)	rs2227310(10)	2.70373	2.03556	0.1537
rs17090911	rs506601(11)	0.771371	0.210723	0.6462
rs17090911	rs547584(11)	2.07633	1.23449	0.2665
rs17090911	rs672016(11)	0.519881	0.880569	0.348
rs17090911	rs507879(11)	0.486573	1.22749	0.2679
rs17090911	rs2282659(11)	0.402489	2.10914	0.1464
rs17090911	rs3181304(19)	1.40962	0.42489	0.5145
rs2227310(10)	rs506601(11)	1.76568	3.09483	0.07854
rs2227310	rs547584(11)	0.858374	0.172843	0.6776
rs2227310	rs672016(11)	0.837395	0.31308	0.5758
rs2227310	rs507879(11)	1.00771	0.000484	0.9824
rs2227310	rs2282659(11)	1.11905	0.105355	0.7455
rs2227310	rs3181304(19)	0.860497	0.280645	0.5963
rs506601(11)	rs547584(11)	0.806561	0.172772	0.6777
rs506601	rs672016(11)	1.00063	2.21E-06	0.9988
rs506601	rs507879(11)	0.529358	2.17754	0.14
rs506601	rs2282659(11)	0.763929	0.418763	0.5176
rs506601	rs3181304(19)	1.23987	0.498552	0.4801
rs547584(11)	rs672016(11)	0.863507	0.133784	0.7145
rs547584	rs507879(11)	1.47352	0.650569	0.4199
rs547584	rs2282659(11)	1.1109	0.049946	0.8232
rs547584	rs3181304(19)	1.13195	0.097502	0.7548

rs672016(11)	rs507879(11)	1.53528	1.21598	0.2702
rs672016	rs2282659(11)	1.58462	1.4465	0.2291
rs672016	rs3181304(19)	1.00935	0.000831	0.977
rs507879(11)	rs2282659(11)	1.02664	0.00335	0.9538
rs507879	rs3181304(19)	0.784872	0.482715	0.4872
rs2282659(11)	rs3181304(19)	0.818834	0.324664	0.5688

SNP, single nucleotide polymorphism; Chr, Chromosome of single nucleotide polymorphism; OR_INT, Odds ratio for interaction; P, p-value

Table S5 The single SNP association studies of CASP7 in psoriasis cases and controls in GWAS.

ID	SNP ID	Chr ID	Chr Position	P-value	Rank	pHWE (case)	pHWE (control)	Call rate (case)	Call rate (control)	Odds ratio
34782	rs4457708	10	113710233	0.03832	35791	0.03107	0.3636	1	1	0.8732
34783	rs10749143	10	113715870	0.03763	35221	0.03981	0.5	1	1	0.8732
34784	rs11196449	10	113720823	0.03434	32394	0.7336	0.5441	1	0.999294	0.8881
34785	rs11196454	10	113733974	0.04284	39777	0.3063	0.5788	0.999301	0.999294	1.144

Table S6 The single SNP association studies of CASP8 in psoriasis cases and controls in GWAS.

SNP ID	Chr ID	Chr Position	P-value	Rank	pHWE (case)	pHWE (control)	Call rate (case)	Call rate (control)	Odds ratio
rs13402616	2	201255044	0.03893	36339	0.8491	0.8177	0.999301	0.999294	1.253
rs16836969	2	201253542	0.0279	26729	1	1	1	1	1.408
rs6743518	2	201231548	0.04506	41633	1	0.3983	1	0.999294	1.345

GWAS, Genome-wide association study
