# Supplementary material for: Association Study of the Caspase Gene Family and Psoriasis Vulgaris Susceptibility in Northeastern China
Source: Biomed Res Int. 2019 Feb 17;2019:2417612. doi: 10.1155/2019/2417612 (PMC6398065; doi:10.1155/2019/2417612)
Supplement: Supplementary Materials — The supplementary materials contain several experimental data and experimental results that are closely related to the content of the manuscript. They are relatively unimportant or large or negative results, but they are still very meaningful for manuscripts. [file 2417612.f1.zip › 2417612.f1/Source file of the final accepted Supplementary material_BMRI_2661540.docx]

Table S1 Genotyping primers.

| Gene | SNP | PCR primer (5’-3’) | PCR primer (3’-5’) | Gene |
| --- | --- | --- | --- | --- |
| CASP1 | rs2282659 | GCAGGAGCGGGGTGAAACTA | GACATCCCACAATGGGCTCTG | 320 |
| CASP3 | rs2705897 | CCATGGCTCAGAAGCACACAAA | CGTGCCCCAGGTTAGGTTAAGA | 192 |
|  | rs4647610 | TGGAGGATCTCGGATGCCTTTT | TGAGCATCGGTATAAGCCCTGAA | 299 |
| CASP4 | rs547584 | TCCATCTGTGACATCTTAGTCTATTCACCA | AAGCCAGATCGCGTGGTTCTATG | 167 |
|  | rs672016 | GGACAGGGGCCATGAACTACA | TGCACATTGGGTTGGCAGTG | 362 |
| CASP5 | rs507879 | CTGCATGGGCCTTGGAGTTG | GCAAAACACGATGTTCTGACATTGA | 242 |
| CASP6 | rs5030545 | CCTCCTGGAACTGCCCCAAG | TGTGCTCACCTAGGGCAAAGG | 313 |
| CASP7 | rs17090911 | TGCCACTGCAACTCCCATCTAA | AGGGTGACGCTGGGTTTTCTTC | 228 |
|  | rs2227310 | CCTGGTTTGTGCAAGCCCTCT | TTGGTGAGCATGGAGACCACAC | 175 |
| CASP8 | rs6704688 | TCGTGCCTCAAGGAGAGGAGAA | TGATTCAGTCATCGTAGATTGGAAGG | 387 |
|  | rs2293554 | GGGTTGAATGGACAGCCTCTGA | TTCCCAAAGCCTCCCAAGTGAT | 230 |
| CASP9 | rs4233532 | TCAGGCAGAGGAGAGGCAAGTG | GCCCACACCCAGTGACATCTTT | 285 |
|  | rs1052576 | TGGCTCCCAAGAAAACAACAGG | CCTATCCGTGCTTCTGGCTCAC | 289 |
| CASP10 | rs12613347 | TTTCAGTGCCCACATTTGCAGT | ACCCGCCACCTCATATTGTTCA | 265 |
|  | rs13006529 | TCGAAGAGTGGACAAACAGGGAAC | AGGGTGACGCTGGGTTTTCTTC | 172 |
| CASP12 | rs506601 | TCCCAGCCTTGTCAGCAGAGAT | TGTGGGACCAAAGATGAAAAGCATA | 439 |
| CASP14 | rs3181304 | GAGCTGCCAGTTCAGCCATGAG | CACACACACAGCAAGTGCTGTGA | 346 |

Table S2 iMLDR probe sequences.

| Gene | Snp Allele | Primer (5’-3’) | LDR product |
| --- | --- | --- | --- |
| CASP1 | rs2282659_modify: | CTCCAAAACTCTTTTGGAAAGAAGAGCTTTTTTTTTTTTTTTTTTTTTT | 64.78 |
|  | rs2282659_A: | TACGGTTATTCGGGCTCCTGTCTGGGTTTGTCCACTCTCCAAACAA | 66.69 |
|  | rs2282659_G: | TTCCGCGTTCGGACTGATATCTGGGTTTGTCCACTCTCCAAACAG | 66.41 |
| CASP3 | rs2705897_modify: | AAAGACATTTGCAAATAAAAGGAAAAAAATTTTTTTTTTTTTTTTTTTTTTT | 63.19 |
|  | rs2705897_G: | TCTCTCGGGTCAATTCGTCCTTTCCTTTTGCTGTGATCTTCTTTAGAAACAGG | 68.02 |
|  | rs2705897_T: | TGTTCGTGGGCCGGATTAGTTCCTTTTGCTGTGATCTTCTTTAGAAACAGT | 66.22 |
| CASP3 | rs4647610_modify: | AGTAGCATTTATTCATTTGATATTTGCACCTTTTTTTTTTTTTTTTTTTTTTTTTT | 63.47 |
|  | rs4647610_C: | TTCCGCGTTCGGACTGATATGGAAGACTGAAACTGCCAAAACTCAGAGTC | 68.69 |
|  | rs4647610FT: | TACGGTTATTCGGGCTCCTGTGGAAGACTGAAACTGCCAAAACTCAGAGTT | 68.18 |
| CASP4 | rs547584_modify: | TAATATGCTTTAAATTAGGTTTTACAAAGATCACATTTTTTTTTTTTTTTTTTTTTT | 63.20 |
|  | rs547584_C: | TCTCTCGGGTCAATTCGTCCTTGGAAGCCTTTCTAGTGCTTTAGAAAAAAGTAG | 66.24 |
|  | rs547584_T: | TGTTCGTGGGCCGGATTAGTGGAAGCCTTTCTAGTGCTTTAGAAAAAAGCAA | 66.45 |
| CASP4 | rs672016_modify: | TAGCCCAAGTAGCCGCCTAGTTTTTTTTTTTT | 63.44 |
|  | rs672016_C: | TACGGTTATTCGGGCTCCTGTTCCAGCGATGGATCCAGATGC | 67.02 |
|  | rs672016_G: | TTCCGCGTTCGGACTGATATTCCAGCGATGGATCCAGATGG | 66.87 |
| CASP5 | rs507879_modify: | ATCATAATATTTTTTCTTTTCCTCTTCCTTCATTTTTTTTTTTTTTTTTTTTTTTTTT | 63.53 |
|  | rs507879_C: | TTCCGCGTTCGGACTGATATCAGGGCCTTGTCTTCAATTTTTGC | 68.70 |
|  | rs507879_T: | TACGGTTATTCGGGCTCCTGTCAGGGCCTTGTCTTCAATTTTCGT | 66.44 |
| CASP6 | rs5030545_modify: | GGGAGGACCAGTTGGAGAGGTTTTTTT | 63.30 |
|  | rs5030545_C: | TTCCGCGTTCGGACTGATATGCTTTCTCATCTGCCATGCATCG | 70.38 |
|  | rs5030545_T: | TACGGTTATTCGGGCTCCTGTGCTTTCTCATCTGCCATGCACCA | 69.00 |
| CASP7 | rs17090911_modify: | CTGATCYGGTGAGTTGTTACCCTTTTTTTT | 63.64 |
|  | rs17090911_A: | TGTTCGTGGGCCGGATTAGTCGTCCACCYGCCCGTGTCAT | 72.85 |
|  | rs17090911_G: | TCTCTCGGGTCAATTCGTCCTTCGTCCACCYGCCCGTGTCAC | 73.59 |
| CASP7 | rs2227310_modify: | CTGGAAATCATGCARATCCTCACTTTTTTTTTTT | 63.13 |
|  | rs2227310_C: | TACGGTTATTCGGGCTCCTGTCCTGGAGGAGCACGGAAAACAC | 66.54 |
|  | rs2227310_G: | TTCCGCGTTCGGACTGATATCCTGGAGGAGCACGGAAAACAG | 66.61 |
| CASP8 | rs6704688_modify: | TAGTTAGGCTAAGTAAGAAAGAGAGAAGACTCAAATTTTTTTTTTTTTTTTTTTTTT | 63.69 |
|  | rs6704688_C: | TTCCGCGTTCGGACTGATATGTTTTTTGAAAAGATAAACAAAATCAACAAATACC | 67.81 |
|  | rs6704688_T: | TACGGTTATTCGGGCTCCTGTGTTTTTTGAAAAGATAAACAAAATCAACAAATGCT |  |
| CASP8 | rs2293554_modify: | AAGCYCCAATCAGCTCAGAGGTTTTTTTTTTTTTTT | 65.10 |
|  | rs2293554_G: | TTCCGCGTTCGGACTGATATGGCACTAGGCAGGGTACCACAAACAC | 68.42 |
|  | rs2293554_T: | TACGGTTATTCGGGCTCCTGTGGCACTAGGCAGGGTACCACAAACAA | 68.71 |
| CASP9 | rs4233532_modify: | GCGCATCCTTGCCCGCTCAGTTTTTTT | 72.65 |
|  | rs4233532_C: | TTCCGCGTTCGGACTGATATGGCGCAGGCCTCCTTGCAGC | 3.77 |
|  | rs4233532_T: | TACGGTTATTCGGGCTCCTGTGGCGCAGGCCTCCTTGCTGT | 71.33 |
| CASP9 | rs1052576_modify: | GCAGGACCACGGTGCTCTGGTTTTTTT | 70.08 |
|  | rs1052576_T: | TGTTCGTGGGCCGGATTAGTGCTTTGCTGGAGCTGGCTCA | 68.80 |
|  | rs1052576_C: | TCTCTCGGGTCAATTCGTCCTTGCTTTGCTGGAGCTGGCACG | 66.35 |
| CASP10 | rs12613347_modify: | GCTCACAAATTTTCCAGTGTCTAGGTTTTTTTTTTTTTTTTTTTTT | 63.04 |
|  | rs12613347_C: | TTCCGCGTTCGGACTGATATTCTCTCCCAAGTTGGGGCAACC | 68.77 |
|  | rs12613347_T: | TACGGTTATTCGGGCTCCTGTTCTCTCCCAAGTTGGGGCAGCT | 66.45 |
| CASP10 | rs13006529_modify: | TATAGCAGAGAGTTTTTGTTGGTTCTTAGACCTTTTTTTTTTTTTTTTTTTTT | 64.92 |
|  | rs13006529_A: | TCTCTCGGGTCAATTCGTCCTTTGCCCCTGGATGCACTTTGAA | 68.12 |
|  | rs13006529_T: | TGTTCGTGGGCCGGATTAGTTGCCCCTGGATGCACTTTGAT | 67.10 |
| CASP12 | rs506601_modify: | CTTCACAATGTGTGACAAACATTTTAAAATTTTTTTTTTTTTTTTTTTTTTTT | 63.25 |
|  | rs506601_A: | TTCCGCGTTCGGACTGATATCTTAAAATTATATTATTGATAATTTGCTTTATTGAA | 58.43 |
|  | rs506601_T: | TACGGTTATTCGGGCTCCTGTCTTAAAATTATATTATTGATAATTTGCTTTATTGAT | 57.85 |
| CASP14 | rs3181304_modify: | CCATTGCTGTTAAGCACCTAGAGCTTTTTTTTTTTTTTTTTTTTTT | 63.47 |
|  | rs3181304_A: | TACGGTTATTCGGGCTCCTGTGGGATCCTAAAACCTACCTGGGACA | 66.27 |
|  | rs3181304_G: | TTCCGCGTTCGGACTGATATGGGATCCTAAAACCTACCTGGGACG | 67.56 |

Table S3. Non-significant results of intergenic interaction analysis of all samples

| SNP(Chr) | SNP(Chr) | OR_INT | Chi-square | P |
| --- | --- | --- | --- | --- |
| rs12613347(2) | rs672016(11) | 1.87088 | 6.89948 | 0.008622 |
| rs13006529(2) | rs506601(11) | 0.380435 | 6.67732 | 0.009765 |
| rs13006529(2) | rs4647610(4) | 3.02893 | 6.06429 | 0.01379 |
| rs4233532(1) | rs13006529(2) | 0.433298 | 4.97048 | 0.02578 |
| rs6704688(2) | rs4647610(4) | 0.54489 | 4.64764 | 0.0311 |
| rs12613347(2) | rs2282659(11) | 0.413222 | 4.62179 | 0.03157 |
| rs506601(11) | rs2282659(11) | 0.433815 | 4.58316 | 0.03229 |
| rs6704688(2) | rs672016(11) | 0.579687 | 4.53809 | 0.03315 |
| rs13006529(2) | rs2227310(10) | 1.90046 | 3.58744 | 0.05822 |
| rs2227310(10) | rs2282659(11) | 0.54177 | 3.31672 | 0.06858 |
| rs4647610(4) | rs672016(11) | 0.632569 | 3.03089 | 0.08169 |
| rs17090911(10) | rs2282659(11) | 0.589216 | 2.83441 | 0.09226 |
| rs13006529(2) | rs17090911(10) | 1.67234 | 2.76846 | 0.09614 |
| rs4647610(4) | rs2282659(11) | 0.520418 | 2.57828 | 0.1083 |
| rs13006529(2) | rs2282659(4) | 0.307957 | 2.41556 | 0.1201 |
| rs2227310(10) | rs547584(11) | 1.44416 | 2.39628 | 0.1216 |
| rs12613347(2) | rs6704688(2) | 0.676949 | 2.25926 | 0.1328 |
| rs506601(11) | rs507879(11) | 0.695608 | 2.20244 | 0.1378 |
| rs506601(11) | rs547584(11) | 0.639831 | 2.09245 | 0.148 |
| rs672016(11) | rs507879(11) | 0.691495 | 2.03727 | 0.1535 |
| rs1052576(1) | rs17090911(10) | 0.771038 | 2.01856 | 0.1554 |
| rs672016(11) | rs2282659(11) | 0.61121 | 1.91344 | 0.1666 |
| rs4233532(1) | rs2227310(10) | 1.28171 | 1.84207 | 0.1747 |
| rs5030545(4) | rs547584(11) | 0.730925 | 1.82892 | 0.1763 |
| rs4233532(1) | rs2705897(4) | 0.763966 | 1.68777 | 0.1939 |
| rs4233532(1) | rs4647610(4) | 1.33038 | 1.5287 | 0.2163 |
| rs4233532(1) | rs672016(11) | 0.742534 | 1.51538 | 0.2183 |
| rs2293554(2) | rs3181304(19) | 0.77683 | 1.49299 | 0.2218 |
| rs1052576(1) | rs13006529(2) | 1.45051 | 1.4818 | 0.2235 |
| rs12613347(2) | rs506601(11) | 0.756185 | 1.47068 | 0.2252 |
| rs13006529(2) | rs547584(11) | 1.72357 | 1.46928 | 0.2255 |
| rs12613347(4) | rs4647610(4) | 0.725222 | 1.46773 | 0.2257 |
| rs4647610(4) | rs507879(11) | 0.710117 | 1.3907 | 0.2383 |
| rs1052576(1) | rs6704688(2) | 1.26616 | 1.37666 | 0.2407 |
| rs6704688(2) | rs17090911(10) | 1.26762 | 1.3755 | 0.2409 |
| rs1052576(1) | rs2282659(11) | 0.695662 | 1.36046 | 0.2435 |
| rs507879(11) | rs3181304(19) | 1.33232 | 1.24832 | 0.2639 |
| rs6704688(2) | rs2282659(11) | 0.639381 | 1.23578 | 0.2663 |
| rs5030545(4) | rs2282659(11) | 1.36171 | 1.11458 | 0.2911 |
| rs2227310(10) | rs672016(11) | 0.805169 | 1.10649 | 0.2928 |
| rs13006529(2) | rs2705897(4) | 1.4222 | 0.948353 | 0.3301 |
| rs13006529(2) | rs2293554(2) | 1.52215 | 0.946049 | 0.3307 |
| rs1052576(1) | rs507879(11) | 1.2219 | 0.939305 | 0.3325 |
| rs2227310(10) | rs506601(11) | 0.826337 | 0.937518 | 0.3329 |
| rs2293554(2) | rs5030545(4) | 1.1738 | 0.934169 | 0.3338 |
| rs4233532(1) | rs506601(11) | 1.20323 | 0.925688 | 0.336 |
| rs12613347(2) | rs547584(11) | 0.780187 | 0.92355 | 0.3365 |
| rs506601(11) | rs3181304(19) | 0.783453 | 0.884717 | 0.3469 |
| rs6704688(2) | rs2227310(10) | 0.811948 | 0.84905 | 0.3568 |
| rs5030545(4) | rs4647610(4) | 1.22329 | 0.820584 | 0.365 |
| rs2293554(2) | rs547584(11) | 1.22551 | 0.779388 | 0.3773 |
| rs507879(11) | rs2282659(11) | 0.699858 | 0.764556 | 0.3819 |
| rs17090911(10) | rs507879(11) | 1.19558 | 0.755069 | 0.3849 |
| rs2705897(4) | rs4647610(4) | 1.25383 | 0.722399 | 0.3954 |
| rs2293554(2) | rs2282659(11) | 1.29398 | 0.714354 | 0.398 |
| rs2705897(4) | rs547584(11) | 0.797241 | 0.691812 | 0.4055 |
| rs17090911(10) | rs547584(11) | 1.22151 | 0.691342 | 0.4057 |
| rs6704688(2) | rs2705897(4) | 0.809578 | 0.671583 | 0.4125 |
| rs6704688(2) | rs5030545(4) | 0.840171 | 0.66854 | 0.4136 |
| rs547584(11) | rs672016(11) | 0.814932 | 0.667406 | 0.414 |
| rs12613347(2) | rs507879(11) | 0.810081 | 0.657286 | 0.4175 |
| rs4233532(1) | rs6704688(2) | 1.1792 | 0.636437 | 0.425 |
| rs2293554(2) | rs672016(11) | 1.17678 | 0.615379 | 0.4328 |
| rs6704688(2) | rs547584(11) | 0.813053 | 0.577134 | 0.4474 |
| rs506601(11) | rs672016(11) | 0.840128 | 0.576201 | 0.4478 |
| rs2227310(10) | rs3181304(19) | 0.84311 | 0.574135 | 0.4486 |
| rs6704688(2) | rs3181304(19) | 1.20849 | 0.565634 | 0.452 |
| rs1052576(1) | rs547584(11) | 1.18922 | 0.562924 | 0.4531 |
| rs547584(11) | rs2282659(11) | 0.758866 | 0.498677 | 0.4801 |
| rs2705897(4) | rs672016(11) | 0.825742 | 0.492009 | 0.483 |
| rs1052576(1) | rs2705897(4) | 1.14044 | 0.490512 | 0.4837 |
| rs4233532(1) | rs507879(11) | 1.15808 | 0.464579 | 0.4955 |
| rs12613347(2) | rs3181304(19) | 1.17661 | 0.455737 | 0.4996 |
| rs2705897(4) | rs2227310(10) | 1.15699 | 0.449123 | 0.5028 |
| rs17090911(10) | rs672016(11) | 0.873979 | 0.445031 | 0.5047 |
| rs2293554(2) | rs506601(11) | 1.13376 | 0.412371 | 0.5208 |
| rs1052576(1) | rs672016(11) | 0.875703 | 0.391196 | 0.5317 |
| rs5030545(4) | rs507879(11) | 0.880494 | 0.353504 | 0.5521 |
| rs1052576(1) | rs2227310(10) | 1.1122 | 0.336675 | 0.5618 |
| rs2282659(11) | rs3181304(19) | 1.26445 | 0.328156 | 0.5667 |
| rs13006529(2) | rs3181304(19) | 1.24908 | 0.323238 | 0.5697 |
| rs5030545(4) | rs506601(11) | 0.901631 | 0.317042 | 0.5734 |
| rs5030545(4) | rs2227310(10) | 0.909314 | 0.306921 | 0.5796 |
| rs13006529(2) | rs6704688(2) | 0.802571 | 0.304457 | 0.5811 |
| rs2705897(4) | rs507879(11) | 0.869665 | 0.277022 | 0.5987 |
| rs2705897(4) | rs2282659(11) | 1.19926 | 0.262991 | 0.6081 |
| rs6704688(2) | rs2293554(2) | 0.900894 | 0.248531 | 0.6181 |
| rs5030545(4) | rs672016(11) | 0.909785 | 0.242418 | 0.6225 |
| rs13006529(2) | rs5030545(4) | 0.858009 | 0.229101 | 0.6322 |
| rs672016(11) | rs3181304(19) | 1.11736 | 0.219557 | 0.6394 |
| rs4647610(4) | rs17090911(10) | 0.907477 | 0.209144 | 0.6474 |
| rs6704688(2) | rs506601(11) | 0.899519 | 0.208401 | 0.648 |
| rs2293554(2) | rs507879(11) | 0.910629 | 0.198247 | 0.6561 |
| rs4233532(1) | rs3181304(19) | 1.09429 | 0.192432 | 0.6609 |
| rs13006529(2) | rs672016(11) | 1.18713 | 0.192105 | 0.6612 |
| rs4233532(1) | rs12613347(2) | 0.890036 | 0.174265 | 0.6763 |
| rs2705897(4) | rs3181304(19) | 0.904446 | 0.167317 | 0.6825 |
| rs6704688(2) | rs507879(11) | 0.899691 | 0.154929 | 0.6939 |
| rs1052576(1) | rs2293554(2) | 1.06852 | 0.152535 | 0.6961 |
| rs12613347(2) | rs13006529(2) | 1.1629 | 0.141733 | 0.7066 |
| rs2293554(2) | rs4647610(4) | 0.922943 | 0.140313 | 0.708 |
| rs2705897(4) | rs17090911(10) | 1.06805 | 0.132593 | 0.7158 |
| rs4233532(1) | rs1052576(1) | 1.06596 | 0.132095 | 0.7163 |
| rs4233532(1) | rs547584(11) | 0.919935 | 0.130972 | 0.7174 |
| rs2293554(2) | rs17090911(10) | 1.06189 | 0.130084 | 0.7183 |
| rs1052576(1) | rs12613347(2) | 0.926385 | 0.122169 | 0.7267 |
| rs12613347(2) | rs2227310(10) | 0.930578 | 0.119546 | 0.7295 |
| rs547584(11) | rs3181304(19) | 0.912289 | 0.105917 | 0.7448 |
| rs4647610(4) | rs506601(11) | 0.928054 | 0.086357 | 0.7689 |
| rs1052576(1) | rs3181304(19) | 1.05884 | 0.078827 | 0.7789 |
| rs4233532(1) | rs5030545(4) | 1.04713 | 0.076873 | 0.7816 |
| rs13006529(2) | rs507879(11) | 0.894779 | 0.07357 | 0.7862 |
| rs1052576(1) | rs5030545(4) | 1.04652 | 0.072894 | 0.7872 |
| rs4647610(4) | rs547584(11) | 0.92635 | 0.068305 | 0.7938 |
| rs2293554(2) | rs2227310(10) | 1.04372 | 0.056335 | 0.8124 |
| rs2293554(2) | rs2705897(4) | 0.956494 | 0.055674 | 0.8135 |
| rs547584(11) | rs507879(11) | 0.943973 | 0.043625 | 0.8346 |
| rs12613347(2) | rs2293554 | 1.04787 | 0.04347 | 0.8348 |
| rs4233532(1) | rs17090911 | 1.03513 | 0.038046 | 0.8454 |
| rs4647610(4) | rs2227310 | 1.0509 | 0.03661 | 0.8483 |
| rs5030545(4) | rs3181304(19) | 1.03532 | 0.029558 | 0.8635 |
| rs17090911(10) | rs2227310 | 0.972697 | 0.023296 | 0.8787 |
| rs4647610(4) | rs3181304(19) | 1.04422 | 0.022803 | 0.88 |
| rs2227310(10) | rs507879(11) | 1.03331 | 0.020342 | 0.8866 |
| rs17090911(10) | rs3181304(19) | 1.02568 | 0.016447 | 0.898 |
| rs12613347(2) | rs17090911(10) | 0.975234 | 0.014048 | 0.9057 |
| rs2705897(4) | rs506601(11) | 1.02578 | 0.013461 | 0.9076 |
| rs5030545(4) | rs2705897(4) | 1.02122 | 0.012416 | 0.9113 |
| rs12613347(2) | rs2705897(4) | 0.973098 | 0.008896 | 0.9249 |
| rs12613347(2) | rs5030545(4) | 1.01575 | 0.006178 | 0.9373 |
| rs1052576(1) | rs506601(11) | 0.985625 | 0.006125 | 0.9376 |
| rs4233532(1) | rs2282659(11) | 1.01487 | 0.001887 | 0.9653 |
| rs1052576(1) | rs4647610(4) | 0.993118 | 0.001114 | 0.9734 |
| rs17090911(10) | rs506601(11) | 1.00553 | 0.000881 | 0.9763 |
| rs4233532(1) | rs2293554(2) | 1.00453 | 0.000687 | 0.9791 |
| rs5030545(4) | rs17090911(10) | 0.997939 | 0.000149 | 0.9902 |

SNP, single nucleotide polymorphism; Chr, Chromosome of single nucleotide polymorphism; OR_INT, Odds ratio for interaction; P, p-value

Table S4. Non-significant results of inter-gene interaction analysis of samples less than 40 years old

| SNP1 | SNP2 | OR_INT | Chi-square | P |
| --- | --- | --- | --- | --- |
| rs4233532(1) | rs1052576(1) | 0.500484 | 4.95215 | 0.02606 |
| rs4233532 | rs12613347(2) | 0.73771 | 1.0474 | 0.3061 |
| rs4233532 | rs13006529(2) | 1.56987 | 1.25533 | 0.2625 |
| rs4233532 | rs6704688(2) | 1.29167 | 0.464027 | 0.4957 |
| rs4233532 | rs2293554(2) | 0.879576 | 0.193231 | 0.6602 |
| rs4233532 | rs5030545(4) | 0.732309 | 0.278148 | 0.5979 |
| rs4233532 | rs2705897(4) | 0.754532 | 0.509995 | 0.4751 |
| rs4233532 | rs4647610(4) | 1.14684 | 0.172763 | 0.6777 |
| rs4233532 | rs17090911(10) | 1.91367 | 1.82988 | 0.1761 |
| rs4233532 | rs2227310(10) | 1.81878 | 3.9662 | 0.04642 |
| rs4233532 | rs506601(11) | 1.07334 | 0.048857 | 0.8251 |
| rs4233532 | rs547584(11) | 1.26819 | 0.340463 | 0.5596 |
| rs4233532 | rs672016(11) | 1.01436 | 0.001817 | 0.966 |
| rs4233532 | rs507879(11) | 1.25217 | 0.408887 | 0.5225 |
| rs4233532 | rs2282659(11) | 1.17881 | 0.238953 | 0.625 |
| rs4233532 | rs3181304(19) | 1.13988 | 0.202368 | 0.6528 |
| rs1052576(1) | rs12613347(2) | 0.734111 | 1.1166 | 0.2907 |
| rs1052576 | rs13006529(2) | 1.51724 | 1.00963 | 0.315 |
| rs1052576 | rs6704688(2) | 1.28636 | 0.426095 | 0.5139 |
| rs1052576 | rs2293554(2) | 0.824896 | 0.410874 | 0.5215 |
| rs1052576 | rs5030545(4) | 0.882317 | 0.04303 | 0.8357 |
| rs1052576 | rs2705897(4) | 0.940507 | 0.026431 | 0.8709 |
| rs1052576 | rs4647610(4) | 1.2279 | 0.354878 | 0.5514 |
| rs1052576 | rs17090911(10) | 1.49908 | 0.786605 | 0.3751 |
| rs1052576 | rs2227310(10) | 1.74155 | 3.31136 | 0.0688 |
| rs1052576 | rs506601(11) | 1.1119 | 0.107367 | 0.7432 |
| rs1052576 | rs547584(11) | 1.18366 | 0.177944 | 0.6731 |
| rs1052576 | rs672016(11) | 1.06869 | 0.036367 | 0.8488 |
| rs1052576 | rs507879(11) | 1.22373 | 0.316181 | 0.5739 |
| rs1052576 | rs2282659(11) | 1.1632 | 0.196556 | 0.6575 |
| rs1052576 | rs3181304(19) | 0.956704 | 0.023143 | 0.8791 |
| rs12613347(2) | rs13006529(2) | 1.07681 | 0.018346 | 0.8923 |
| rs12613347 | rs6704688(2) | 0.711423 | 0.558274 | 0.455 |
| rs12613347 | rs2293554(2) | 0.714423 | 1.20181 | 0.273 |
| rs12613347 | rs5030545(4) | 1.39516 | 0.27832 | 0.5978 |
| rs12613347 | rs2705897(4) | 1.35253 | 0.501609 | 0.4788 |
| rs12613347 | rs4647610(4) | 0.968516 | 0.010871 | 0.917 |
| rs12613347 | rs17090911(10) | 0.43267 | 2.0747 | 0.1498 |
| rs12613347 | rs2227310(10) | 0.979232 | 0.006084 | 0.9378 |
| rs12613347 | rs506601(11) | 1.31798 | 0.69494 | 0.4045 |
| rs12613347 | rs547584(11) | 0.748639 | 0.533255 | 0.4652 |
| rs12613347 | rs672016(11) | 1.42123 | 1.1606 | 0.2813 |
| rs12613347 | rs507879(11) | 1.57541 | 1.38588 | 0.2391 |
| rs12613347 | rs2282659(11) | 1.47615 | 1.11482 | 0.291 |
| rs12613347 | rs3181304(19) | 1.11002 | 0.131173 | 0.7172 |
| rs13006529(2) | rs6704688(2) | 4.84722 | 8.89996 | 0.002852 |
| rs13006529 | rs2293554(2) | 0.879058 | 0.074297 | 0.7852 |
| rs13006529 | rs5030545(4) | 0.534802 | 0.969065 | 0.3249 |
| rs13006529 | rs2705897(4) | 0.419086 | 3.33315 | 0.0679 |
| rs13006529 | rs4647610(4) | 0.59357 | 1.82545 | 0.1767 |
| rs13006529 | rs17090911(10) | 0.431271 | 1.65213 | 0.1987 |
| rs13006529 | rs2227310(10) | 1.34796 | 0.579206 | 0.4466 |
| rs13006529 | rs506601(11) | 0.723868 | 0.590365 | 0.4423 |
| rs13006529 | rs547584(11) | 0.906464 | 0.04475 | 0.8325 |
| rs13006529 | rs672016(11) | 1.32243 | 0.422269 | 0.5158 |
| rs13006529 | rs507879(11) | 0.341075 | 3.14966 | 0.07594 |
| rs13006529 | rs2282659(11) | 0.34197 | 3.92804 | 0.04749 |
| rs13006529 | rs3181304(19) | 0.829898 | 0.315742 | 0.5742 |
| rs6704688(2) | rs2293554(2) | 0.768402 | 0.325137 | 0.5685 |
| rs6704688 | rs5030545(4) | 0.872099 | 0.073418 | 0.7864 |
| rs6704688 | rs2705897(4) | 0.492185 | 2.2981 | 0.1295 |
| rs6704688 | rs4647610(4) | 0.604051 | 1.76515 | 0.184 |
| rs6704688 | rs17090911(10) | 0.687349 | 0.320075 | 0.5716 |
| rs6704688 | rs2227310(10) | 1.62567 | 1.69793 | 0.1926 |
| rs6704688 | rs506601(11) | 1.08835 | 0.043105 | 0.8355 |
| rs6704688 | rs547584(11) | 1.08547 | 0.032909 | 0.856 |
| rs6704688 | rs672016(11) | 0.836206 | 0.181155 | 0.6704 |
| rs6704688 | rs507879(11) | 0.390537 | 2.52758 | 0.1119 |
| rs6704688 | rs2282659(11) | 0.339471 | 4.27468 | 0.03868 |
| rs6704688 | rs3181304(19) | 1.05008 | 0.022479 | 0.8808 |
| rs2293554(2) | rs5030545(4) | 0.947435 | 0.008905 | 0.9248 |
| rs2293554 | rs2705897(4) | 1.53725 | 0.826111 | 0.3634 |
| rs2293554 | rs4647610(4) | 2.45224 | 5.22077 | 0.02232 |
| rs2293554 | rs17090911(10) | 1.75171 | 1.02167 | 0.3121 |
| rs2293554 | rs2227310(10) | 0.653939 | 2.06846 | 0.1504 |
| rs2293554 | rs506601(11) | 0.896776 | 0.105223 | 0.7456 |
| rs2293554 | rs547584(11) | 0.746584 | 0.482849 | 0.4871 |
| rs2293554 | rs672016(11) | 0.694509 | 0.979045 | 0.3224 |
| rs2293554 | rs507879(11) | 1.14932 | 0.095373 | 0.7575 |
| rs2293554 | rs2282659(11) | 0.952456 | 0.013004 | 0.9092 |
| rs2293554 | rs3181304(19) | 1.19779 | 0.301538 | 0.5829 |
| rs5030545(4) | rs2705897(4) | 0.122696 | 6.1767 | 0.01294 |
| rs5030545 | rs4647610(4) | 0.243778 | 4.60466 | 0.03189 |
| rs5030545 | rs17090911(10) | 0.08234 | 4.04946 | 0.04419 |
| rs5030545 | rs2227310(10) | 0.962213 | 0.0052 | 0.9425 |
| rs5030545 | rs506601(11) | 0.39033 | 2.3118 | 0.1284 |
| rs5030545 | rs547584(11) | 0.631523 | 0.524085 | 0.4691 |
| rs5030545 | rs672016(11) | 1.19673 | 0.070024 | 0.7913 |
| rs5030545 | rs507879(11) | 0.498419 | 0.98531 | 0.3209 |
| rs5030545 | rs2282659(11) | 0.43848 | 1.2635 | 0.261 |
| rs5030545 | rs3181304(19) | 1.6296 | 0.855262 | 0.3551 |
| rs2705897(4) | rs4647610(4) | 0.484373 | 1.98496 | 0.1589 |
| rs2705897 | rs17090911(10) | 2.27607 | 1.35217 | 0.2449 |
| rs2705897 | rs2227310(10) | 0.972657 | 0.005684 | 0.9399 |
| rs2705897 | rs506601(11) | 0.476877 | 2.37964 | 0.1229 |
| rs2705897 | rs547584(11) | 1.33447 | 0.291701 | 0.5891 |
| rs2705897 | rs672016(11) | 1.48406 | 0.679833 | 0.4096 |
| rs2705897 | rs507879(11) | 2.00798 | 1.65796 | 0.1979 |
| rs2705897 | rs2282659(11) | 1.64458 | 0.871117 | 0.3506 |
| rs2705897 | rs3181304(19) | 1.36572 | 0.584167 | 0.4447 |
| rs4647610(4) | rs17090911(10) | 1.42186 | 0.323734 | 0.5694 |
| rs4647610 | rs2227310(10) | 0.816066 | 0.474276 | 0.491 |
| rs4647610 | rs506601(11) | 0.905952 | 0.081215 | 0.7757 |
| rs4647610 | rs547584(11) | 2.90082 | 5.91442 | 0.01502 |
| rs4647610 | rs672016(11) | 0.720905 | 0.764754 | 0.3818 |
| rs4647610 | rs507879(11) | 1.19126 | 0.185494 | 0.6667 |
| rs4647610 | rs2282659(11) | 1.04906 | 0.012767 | 0.91 |
| rs4647610 | rs3181304(19) | 0.72408 | 1.02716 | 0.3108 |
| rs17090911(10) | rs2227310(10) | 2.70373 | 2.03556 | 0.1537 |
| rs17090911 | rs506601(11) | 0.771371 | 0.210723 | 0.6462 |
| rs17090911 | rs547584(11) | 2.07633 | 1.23449 | 0.2665 |
| rs17090911 | rs672016(11) | 0.519881 | 0.880569 | 0.348 |
| rs17090911 | rs507879(11) | 0.486573 | 1.22749 | 0.2679 |
| rs17090911 | rs2282659(11) | 0.402489 | 2.10914 | 0.1464 |
| rs17090911 | rs3181304(19) | 1.40962 | 0.42489 | 0.5145 |
| rs2227310(10) | rs506601(11) | 1.76568 | 3.09483 | 0.07854 |
| rs2227310 | rs547584(11) | 0.858374 | 0.172843 | 0.6776 |
| rs2227310 | rs672016(11) | 0.837395 | 0.31308 | 0.5758 |
| rs2227310 | rs507879(11) | 1.00771 | 0.000484 | 0.9824 |
| rs2227310 | rs2282659(11) | 1.11905 | 0.105355 | 0.7455 |
| rs2227310 | rs3181304(19) | 0.860497 | 0.280645 | 0.5963 |
| rs506601(11) | rs547584(11) | 0.806561 | 0.172772 | 0.6777 |
| rs506601 | rs672016(11) | 1.00063 | 2.21E-06 | 0.9988 |
| rs506601 | rs507879(11) | 0.529358 | 2.17754 | 0.14 |
| rs506601 | rs2282659(11) | 0.763929 | 0.418763 | 0.5176 |
| rs506601 | rs3181304(19) | 1.23987 | 0.498552 | 0.4801 |
| rs547584(11) | rs672016(11) | 0.863507 | 0.133784 | 0.7145 |
| rs547584 | rs507879(11) | 1.47352 | 0.650569 | 0.4199 |
| rs547584 | rs2282659(11) | 1.1109 | 0.049946 | 0.8232 |
| rs547584 | rs3181304(19) | 1.13195 | 0.097502 | 0.7548 |
| rs672016(11) | rs507879(11) | 1.53528 | 1.21598 | 0.2702 |
| rs672016 | rs2282659(11) | 1.58462 | 1.4465 | 0.2291 |
| rs672016 | rs3181304(19) | 1.00935 | 0.000831 | 0.977 |
| rs507879(11) | rs2282659(11) | 1.02664 | 0.00335 | 0.9538 |
| rs507879 | rs3181304(19) | 0.784872 | 0.482715 | 0.4872 |
| rs2282659(11) | rs3181304(19) | 0.818834 | 0.324664 | 0.5688 |

SNP, single nucleotide polymorphism; Chr, Chromosome of single nucleotide polymorphism; OR_INT, Odds ratio for interaction; P, p-value

Table S5 The single SNP association studies of CASP7 in psoriasis cases and controls in GWAS.

| ID | SNP ID | Chr ID | Chr Position | P-value | Rank | pHWE (case) | pHWE (control) | Call rate (case) | Call rate (control) | Odds ratio |
| --- | --- | --- | --- | --- | --- | --- | --- | --- | --- | --- |
| 34782 | rs4457708 | 10 | 113710233 | 0.03832 | 35791 | 0.03107 | 0.3636 | 1 | 1 | 0.8732 |
| 34783 | rs10749143 | 10 | 113715870 | 0.03763 | 35221 | 0.03981 | 0.5 | 1 | 1 | 0.8732 |
| 34784 | rs11196449 | 10 | 113720823 | 0.03434 | 32394 | 0.7336 | 0.5441 | 1 | 0.999294 | 0.8881 |
| 34785 | rs11196454 | 10 | 113733974 | 0.04284 | 39777 | 0.3063 | 0.5788 | 0.999301 | 0.999294 | 1.144 |

Table S6 The single SNP association studies of CASP8 in psoriasis cases and controls in GWAS.

| SNP ID | Chr ID | Chr Position | P-value | Rank | pHWE (case) | pHWE (control) | Call rate (case) | Call rate (control) | Odds ratio |
| --- | --- | --- | --- | --- | --- | --- | --- | --- | --- |
| rs13402616 | 2 | 201255044 | 0.03893 | 36339 | 0.8491 | 0.8177 | 0.999301 | 0.999294 | 1.253 |
| rs16836969 | 2 | 201253542 | 0.0279 | 26729 | 1 | 1 | 1 | 1 | 1.408 |
| rs6743518 | 2 | 201231548 | 0.04506 | 41633 | 1 | 0.3983 | 1 | 0.999294 | 1.345 |

GWAS, Genome-wide association study
